# COX7A1 enhances the sensitivity of human NSCLC cells to cystine deprivation-induced ferroptosis via regulating mitochondrial metabolism

**DOI:** 10.1038/s41419-022-05430-3

**Published:** 2022-11-23

**Authors:** Yetong Feng, Jiayi Xu, Mengjiao Shi, Rongrong Liu, Lei Zhao, Xin Chen, Miaomiao Li, Yaping Zhao, Jiahui Chen, Wenjing Du, Pengfei Liu

**Affiliations:** 1 National & Local Joint Engineering Research Center of Biodiagnosis and Biotherapy, The Second Affiliated Hospital of Xi’an Jiaotong University, Xi’an, China; 2 Core Research Laboratory, The Second Affiliated Hospital of Xi’an Jiaotong University, Xi’an, China; 3 International Joint Research Center on Cell Stress and Disease Diagnosis and Therapy, The Second Affiliated Hospital of Xi’an Jiaotong University, Xi’an, China; 4 Shaanxi Provincial Clinical Research Center for Hepatic & Splenic Diseases, The Second Affiliated Hospital of Xi’an Jiaotong University, Xi’an, China; 5 Ambulatory Surgical Center, The 2nd Clinical medical College (Shenzhen People’s Hospital) of Jinan University, The 1st Affiliated Hospital of Southern University of Science and Technology, Shenzhen, China; 6 Department of Laboratory Medicine, The 2nd Clinical medical College (Shenzhen People’s Hospital) of Jinan University, The 1st Affiliated Hospitals of Southern University of Science and Technology, Shenzhen, China; 7 Department of Regenerative Medicine, School of Pharmaceutical Science, Jilin University, Changchun, China; 8 The Postdoctoral Research Station, School of Medicine, Nankai University, Tianjin, China; 9 Key Laboratory of Environment and Genes Related To Diseases, Xi’an Jiaotong University, Ministry of Education of China, Xi’an, China

**Keywords:** Cancer therapeutic resistance, Metabolic pathways

## Abstract

COX7A1, a subunit of cytochrome c oxidase, holds an important position in the super-assembly which integrates into multi-unit heteromeric complexes peripherally in the mitochondrial electron transport chain (ETC). Recently, some studies indicated the significant potential of COX7A1 in cancer metabolism and therapy. However, the underlying metabolic process and therapy mechanism remain unclear. In this study, COX7A1-overexpressed cell line was established via lentivirus transduction. The relationship between COX7A1 and ferroptosis, a novel form of cell death driven by iron-dependent lipid peroxidation, was further analyzed in different human non-small-cell lung carcinoma (NSCLC) cells respectively. Our results showed that COX7A1 increased the sensitivity of NSCLC cells to the ferroptosis induced by cysteine deprivation via enhancing the tricarboxylic acid (TCA) cycle and the activity of complex IV in mitochondrial ETC. Meanwhile, COX7A1 suppressed mitochondrial dynamics as well as mitochondrial biogenesis and mitophagy through blocking autophagic flux. The autophagy activator, rapamycin, relieved the autophagic blockage and further strengthened the sensitivity to cysteine deprivation-induced ferroptosis of NSCLC cells in vitro and in vivo. Taken together, our data indicate the close association of COX7A1 with cysteine deprivation-induced ferroptosis, and provide a novel insight into the therapy mode against human NSCLC.

## Introduction

Ferroptosis is a novel type of cell death, which is different from apoptosis, necrosis or autophagic cell death [1, 2]. In recent years, lots of research has demonstrated that ferroptosis results from iron-dependent lipid peroxidation. The cellular redox contents are mainly regulated by glutathione (GSH), the substrate of phospholipid hydroperoxidases. Therefore, the inhibition of cystine-glutamate antiporter (system Xc^-^) leads to the depletion of cellular cysteine as well as GSH, which further disrupts redox homeostasis and results in ferroptosis finally. In addition, the inactivation of glutathione peroxidase 4 (GPX4) and ferroptosis suppressor protein 1 (FSP1), which are required for clearance of lipid ROS, also can induce ferroptosis in the cells with normal levels of cysteine and GSH effectively [3–5]. Recently, some scientists demonstrated that the inactivation of DHODH also leads to extensive mitochondrial lipid peroxidation as well as ferroptosis in cancer cells, identifying a novel ferroptosis defence mechanism in mitochondria [6].

Even though the detailed function and mechanism of ferroptosis is still unclear, some studies have showed the relationship between ferroptosis and different diseases, such as brain injury [7–9], lung injury [10], Alzheimer’s disease [11], carcinogenesis [12, 13] and ischemia-reperfusion injury [9]. In the field of cancer research, scientists also notices that some types of cancer cells, which are resistant to traditional chemotherapy, are sensitive to ferroptosis inducers, indicating the promising potential of ferroptosis to become a novel therapeutic approach to treat chemotherapy-resistant cancers [14, 15].

Mitochondria holds a key position in the regulation of cell signaling transduction and cellular metabolism. The most important role of mitochondria is to create energy via oxidative phosphorylation (OXPHOS). In addition, mitochondria also join the metabolism of amino acid and fatty acid, and is the major organelle in iron utilization [16–18]. Moreover, ferroptosis was primarily characterized by the pathological changes of mitochondria, such as the condensed density of mitochondrial membrane and the reduced mitochondrial volume, and mitochondria also become a target for the development of ferroptosis inducers or inhibitors [1, 19, 20]. Therefore, the pertinence between mitochondria and ferroptosis has attracted many scientists’ attention in recent years. In 2019, Gao et al. first investigated the role of mitochondria in ferroptosis. Their results indicated that mitochondria play an important role in the ferroptosis induced by cysteine deprivation, but not in the ferroptosis induced by GPX4 inhibition. Besides, both tricarboxylic acid (TCA) cycle and mitochondrial electron transport chain (ETC) activity are necessary for the generation of lipid ROS in the ferroptosis induced by cysteine deprivation. The inhibition of those canonical metabolic activity of mitochondria can rescue the ferroptosis induced by erastin or cystine starvation in cancer cells [16]. Therefore, the functional relevance of mitochondria in ferroptosis is highly context dependent.

Cytochrome c oxidase (COX) is an important complex in mitochondrial ETC, and its function is to catalyze the conversion of molecular oxygen into water, which is the terminal limiting step of mitochondrial respiration. COX family includes 13 different subunits, and one of them is cytochrome c oxidase subunit 7a (COX7A) [21, 22]. COX7A contains two isoforms, which are encoded by different genes, *COX7A1* and *COX7A2*. Some studies have indicated that COX7A1 holds an important position in the super-assembly which integrates peripherally into multi-unit heteromeric complexes in mitochondrial ETC [23, 24]. Besides the function on energy generation and metabolism, our previous study indicated that COX7A1 could block autophagic flux through the upregulation of NOX2 as well as the downregulation of PGC-1α. Meanwhile, the overexpression of COX7A1 inhibited cell proliferation capacity and colony formation ability of human non-small cell lung cancer cells, which was partly dependent on the regulation of autophagy [25].

Autophagy is a mechanism of dysfunctional components degradation and intracellular protein and organelle recycling. During this self-degradative process, misfolded or aggregated proteins and damaged organelles go into a double-membrane vesicle, autophagosome, then autophagosome further fuses with the lysosome to form autolysosome, resulting in the degradation eventually [26, 27]. The degradation of mitochondria by autophagy is called mitophagy, which is indispensable to eliminate damaged mitochondrial structure and maintain mitochondrial homeostasis [28, 29]. Besides, the interplay between mitochondrial dynamics and mitophagy is also considered as a key mechanism in different mitochondrial disease models, even in ferroptosis regulation [13, 20, 30]. However, the detailed relationship among COX7A1, autophagy, mitochondria and ferroptosis is still unclear. In this study, we mainly explored the role of COX7A1 in ferroptosis regulation in human nonsmall-cell lung carcinoma (NSCLC) model. Our results indicated that COX7A1 enhanced TCA cycle and the activity of complex IV in mitochondrial ETC, which increased the sensitivity of NSCLC cells to the ferroptosis induced by cysteine deprivation. Meanwhile, COX7A1 blocked autophagic flux and inhibited mitochondrial dynamics as well as mitochondrial biogenesis and mitophagy, and autophagy activator relieved the blockage induced by COX7A1 and further promoted the sensitivity to cysteine deprivation-induced ferroptosis in NSCLC cells. Totally, our study elucidates the promising therapeutic position of COX7A1 and autophagy in the treatment of human NSCLC via regulating mitochondria and ferroptosis.

## Materials and methods

### Cell culture

Human NSCLC cell lines, NCI-H838 (H838) and NCI-H1703 (H1703), were purchased from the American Type Culture Collection (ATCC, USA). Both cell lines were cultured using RPMI-1640 medium (Gibco, USA) supplemented with 10% FBS (Gibco, USA), 100 U/mL penicillin and 0.1 g/mL streptomycin (Sigma, USA) in a humidified 37˚C incubator with 5% CO_2_.

To establish COX7A1-overexpressed cell line, the coding sequence of human *COX7A1* gene was amplified using PCR (Phusion® High-Fidelity DNA Polymerase, New England BioLabs, USA) and cloned into a pLenti6/V5-D-TOPO vector (Addgene, USA) subsequently. The empty pLenti6/V5-D-TOPO vector was applied in the Ctrl group. Then, the lentivirus was prepared using ViraPower™ Lentiviral Expression Systems (K495000, Thermo Fisher, USA). After transduction, the stable positive cells were selected by blasticidin treatment (5 µg/ml, Thermo Fisher, USA).

### Cell viability assay

The cell viability was measured in different groups using Cell Counting Kit-8 (CCK-8, Dojindo, Japan) as the references [25, 31]. In brief, 10 μl of the CCK-8 reagent was added to each well (containing 100 μl of medium) on the 96-well microplate, and the microplate was incubated at 37 ˚C for 4 h. Finally, the OD (450 nm) were detected in each group (*n* = 3). The cell viability in the Ctrl group (without any treatment) was regarded as “100%”, and the relative cell viability of the other groups was evaluated respectively.

### Real-time qRT-PCR

Real-time qPCR was then performed as our previously described [10, 32, 33]. Herein, β-actin was applied in the qPCR normalization, and all of experiments were measured in triplicate. Primer sequences (5’-3’) are as follows:

*COX7A1*-Forward 5’-CCGCTTTCAGAACCGAGTG-3’

*COX7A1*-Reverse 5’-CCCTTCAGGTACAACGGGA-3’

*PTGS2*-Forward 5’-CGGTGAAACTCTGGCTAGACAG-3’

*PTGS2*-Reverse 5’-GCAAACCGTAGATGCTCAGGGA-3’

*β-actin*-Forward 5’-CCCAGAGCAAGAGAGG-3’

*β-actin*-Reverse 5’-GTCCAGACGCAGGATG-3’

### Western blot

In this study, the western blot assay was performed as our previously described [34, 35]. Briefly, the protein sample (20 µg/lane) was separated using 8% or 12% SDS-PAGE gel and then transferred to nitrocellulose membranes. The membrane was blocked using 5% bovine serum albumin, then incubated with primary antibodies overnight at 4 ˚C. In the present study, the primary antibodies used were: anti-COX7A1 (1:3000; Abcam, ab123591, USA), anti-p62 (1:3000; Abcam, ab56316, USA), anti-LC3 (1:3000; Sigma, L7543, USA), anti-TMO20 (1:1000; Santa Crus, sc-17764, USA), anti-TIM23 (1:2000; Abcam, ab230253, USA), anti-DRP1 (1:2000; Abcam, ab184247, USA), anti-MFN1 (1:1000; Proteintech, 13798-1-AP, USA), anti-VDAC (1:2000; Proteintech, 10866-1-AP, USA), anti-PINK (1:2000; Abcam, ab137361, USA), anti-PARKIN (1:2000; Abcam, ab77924, USA), anti-PGC-1α (1:3000; Abcam, ab54481, USA), anti-TFAM (1:1000; Cell Signaling, 7495, USA) and anti-GAPDH (1:3000; Santa Cruz, sc-47724, USA). After incubated with HRP-labeled secondary antibodies, the protein bands were visualized using an enhanced chemiluminescence kit (SuperSignal West Femto Maximum Sensitivity Substrate, Thermo Fisher Scientific, USA) as well as ChemiDoc Imagers (Bio-Rad Laboratories, USA). Full-length western blots can be found in Supplemental Materials.

### Live cell immunofluorescence microscopy

The Live cell immunofluorescence microscopy was performed as our previously described [25, 36]. Briefly, H838 cells and H1703 cells, cultured in 35 mm glass-bottom dishes, were transfected with ptf-mRFP-GFP-LC3 reporter construct using Lipofectamine 3000 (Thermo Fisher, USA) for 24 h. Then, the cells in each group were imaged with a Zeiss Observer Fluorescence Microscope (Zeiss, Germany) in phenol red-free medium.

### Evaluation of malondialdehyde (MDA) and 4-hydroxynonenal (4-HNE)

In this study, the MDA and 4-HNE in different groups were measured to evaluate ferroptosis level. The MDA concentration and 4-HNE concentration in cell lysates were assessed using the Lipid Peroxidation (MDA) Assay Kit (MAK085, Sigma-Aldrich, USA), and Lipid Peroxidation (4-HNE) Assay Kit (ab238538, Abcam, USA) according to the manufacturer’s instructions.

### BODIPY staining

For BODIPY staining, H838 cells and H1703 cells were washed with PBS, and incubated with 1 μM BODIPY 581/591 C11 (Thermo Fisher, USA) for 0.5 h at 37 ^o^C. Then the cells were washed with PBS and analyzed with Zeiss Observer Fluorescence Microscope (Zeiss, Germany).

### TCA cycle assay

To evaluate the TCA cycle in each group, the key metabolites, α-Ketoglutarate (α-KG) and succinate (Suc), were measured respectively. In our research, α-KG was detected with a-Ketoglutarate Assay Kit (MAK054, Sigma, USA), and Suc was measured using Succinate Colorimetric Assay Kit (MAK184, Sigma, USA) according to the manufacturer’s instructions.

### Evaluation of mitochondrial ETC activity

Herein, mitochondria in cell samples were isolated using Mitochondria Isolation Kit for Cultured Cells (ab110170, Abcam, USA). Then, the activity of Complex I to IV was evaluated using Complex I Enzyme Activity Assay Kit (ab109721, Abcam, USA), Complex II Enzyme Activity Assay Kit (ab109908, Abcam, USA), Mitochondrial Complex III Activity Assay Kit (K520-100, Biovision, USA), and Complex IV Enzyme Activity Assay Kit (ab109909, Abcam, USA) according to the manufacturer’s instructions respectively.

### Measurement of mitochondrial membrane potential (MMP) and ATP

The presence of mitochondria was evaluated by MitoTracker^®^ Green FM (9074S, Cell signaling, USA) staining. To detect MMP, the cells in different groups were incubated with 0.5 mM TMRE (ab113852, Abcam, USA) for 30 min, then washed with PBS. Finally, the MMP level in each group was analyzed by flow cytometry. In addition, the ATP level was measured using ATP Colorimetric/Fluorometric Assay Kit (MAK190, Sigma, USA) according to the manufacturer’s instructions.

### Xenograft mouse model

NOG mice were purchased from Charles River Laboratories. The 6-week-old male mice (weight = 18–22 g) were injected with cancer cells (100 μL containing 2 × 10^7^ cells/mouse, i.h.). Then, the mice were divided into 4 groups (Ctrl, Rap, Era, and Era+Rap) for WT and COX7A1 overexpressed cells, respectively. The tumor volume was measured using vernier caliper (Volume = π/6 × Length × Width^2^). After tumor volume was about 100–150 mm³, the mice were divided into 4 groups randomly based on tumor volume. Rapmycin (0.5 mg/kg) and erastin (15 mg/kg) were dissolved in 5% DMSO/corn oil, and injected into the mice intraperitoneally (twice in one week) for five weeks. Then, the mice were sacrificed and tumor weight was measured in each group (*n* = 5). The order of treatments and measurements was kept same every time to minimize potential confounders. In addition, the survival rate within 120 days was also evaluated in different groups (*n* = 10). There were no exclusions or blinding design in our study.

### Statistical analysis

In this study, the results were presented as mean ± SD (*n* = 3), and the statistical analysis was performed using SPSS 17.0. The unpaired Student’s t-tests were used to compare the means between two different groups, and one-way ANOVA with Bonferroni’s correction was applied to compare the means among three or more groups. No statistical methods were applied to predetermine the sample size in different groups, and no blinding method or animal exclusion criteria was used for animal work. The variance was similar between groups that were being statistically compared. In addition, the one-tailed test was used in the Student’s t-test, and *p* < 0.05 was considered statistically significant.

## Results

### COX7A1 enhances the sensitivity of NSCLC cells to the ferroptosis induced by cysteine deprivation

**Fig. 1:**
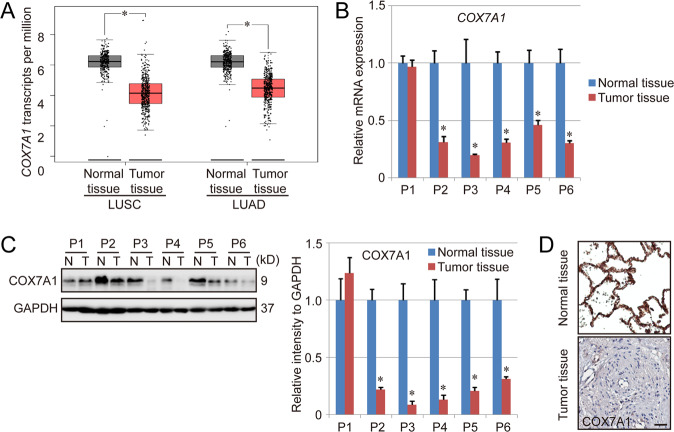
The expression of COX7A1 in normal lung tissues and NSCLC tissues. **A** Comparison of COX7A1 mRNA levels between normal lung tissues and NSCLC tissues. The expression level of COX7A1 in LUAD patients (num (Tumor tissue) = 483; num (Normal tissue) = 347) and LUSC patients (num (Tumor tissue) = 486; num (Normal tissue) = 338) was evaluated using TCGA database. Besides, the tumor tissues and normal tissues from 6 NSCLC patients (Patient 1 (P1) to Patient 6 (P6)) were harvested for COX7A1 detection in both mRNA (**B**) and protein levels (**C**). Results are expressed as mean ± SD, and the P value less than 0.05 was considered statistically significant. *:*P* < 0.05 compared between normal tissue and tumor tissue. **D** Immunohistochemistry staining of COX7A1 in the tumor tissue and normal tissue from Patient 3. Scale bar = 100 μm.

Some studies have indicated the anti-cancer potential of COX7A1 in several types of lung cancer cells. Herein, lung squamous cell carcinoma (LUSC) and lung adenocarcinoma (LUAD), two most major subtypes of NSCLC, were chosen to explore the position of COX7A1. To analyze the relationship between COX7A1 and NSCLC, the expression level of COX7A1 in LUSC tissues and LUAD tissues was evaluated using TCGA database first. It was found that the level of COX7A1 was significantly lower in the tumor tissues compared with normal lung tissues (Fig. 1A). To confirm such results, the tumor tissues and normal tissues from 6 NSCLC patients were harvested for COX7A1 detection. Except Patient 1 (P1), the results from Patient 2 to Patient 6 (P2-P6) also showed that the expression of COX7A1 in tumor tissues was much lower than normal lung tissues in both RNA level and protein level (Fig. 1B–D), indicating the anti-cancer potential of COX7A1 during the process of NSCLC development.

**Fig. 2:**
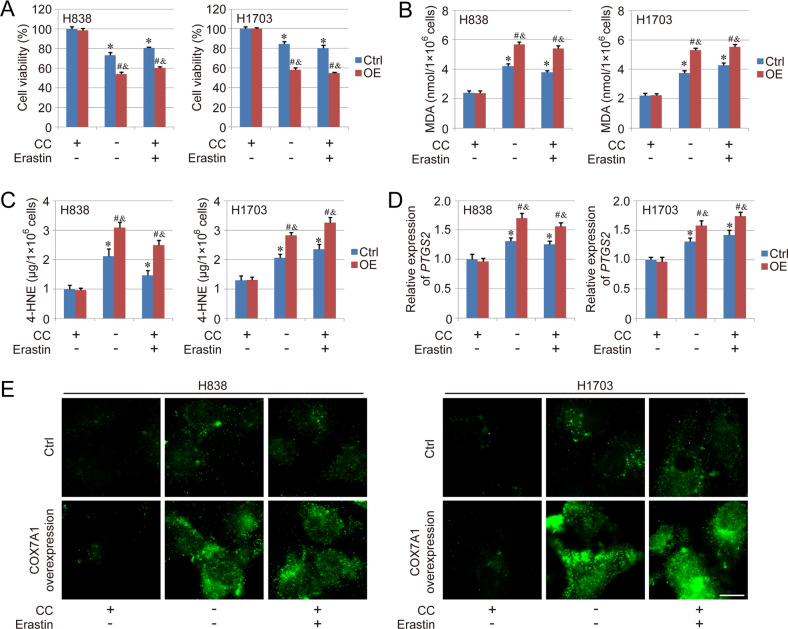
COX7A1 enhances the sensitivity of NSCLC cells to cysteine deprivation-induced ferroptosis. The ferroptosis model in NSCLC cells was induced by cellular cysteine (CC) starvation and the treatment of erastin (5 μM) for 24 h respectively. Then, cell viability was evaluated using CCK-8 method (**A**). To analyze the ferroptosis level, MDA (**B**) and 4-HNE (**C**) levels were measured in different groups. The expression of *PTGS2* (**D**) and lipid peroxidation (**E**) were detected using qPCR and BODIPY staining respectively. Scale bar = 10 μm. Results are expressed as mean ± SD, and the *P* value less than 0.05 was considered statistically significant. *:*P* < 0.05 compared with the Ctrl group in CC(+)Erastin(−) condition. #:*P* < 0.05 compared between Ctrl group and Overexpression (OE) group in same condition. &:*P* < 0.05 compared with the OE group in CC(+)Erastin(−) condition.

To explore the function of COX7A1 in ferroptosis, the COX7A1 overexpression models were established in H838 (human LUAD cell line) and H1703 (human LUSC cell line) respectively, which were confirmed by qPCR and western blot (Figure S1A, B). Similar to our previous study, the overexpression of COX7A1 inhibited cell proliferation capacity weakly (Figure S1C). However, the basal level of MDA, an important maker of ferroptosis, didn’t show obvious difference between Ctrl group and Overexpression group (Figure S1D). Meanwhile, ferroptosis model was induced by cellular cysteine (CC) starvation and the treatment with system Xc^-^ inhibitor, erastin, respectively. We found that the cell viability was inhibited by both CC starvation and erastin treatment, and the inhibition could be further enhanced by COX7A1 overexpression (Fig. 2A). Even though the basal levels of MDA and 4-HNE didn’t show difference between Ctrl group and Overexpression group, CC starvation and erastin treatment induced higher levels of MDA and 4-HNE in COX7A1 overexpression group than Ctrl group (Fig. 2B, C). In addition, the expression of *PTGS2*, a maker gene of ferroptosis, as well as lipid peroxidation, were measured using qPCR and BODIPY staining respectively, and the results also showed a similar tendency as MDA and 4-HNE in different groups (Fig. 2D, E), suggesting that the sensitivity of NSCLC cells to the ferroptosis induced by cysteine deprivation could be enhanced by COX7A1 overexpression.

### COX7A1 promotes mitochondrial TCA cycle in NSCLC cells

**Fig. 3:**
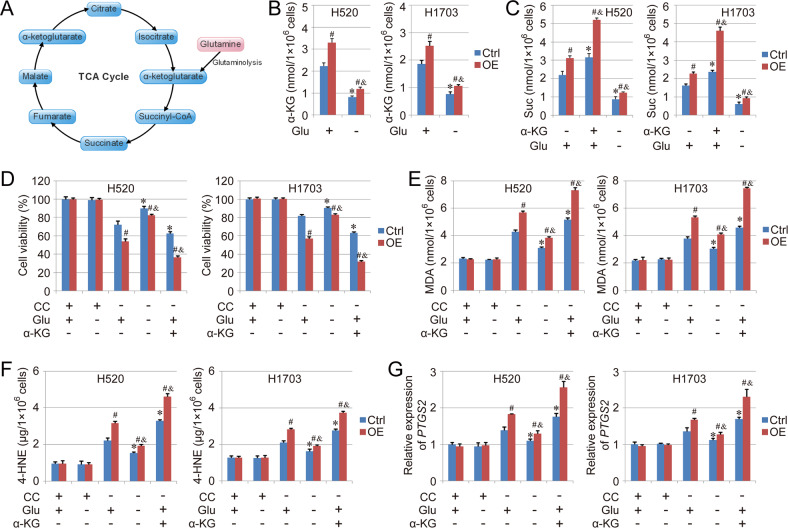
COX7A1 promotes mitochondrial TCA cycle in NSCLC cells. An overview of TCA cycle was showed in **A** The ferroptosis model was induced by cellular cysteine (CC) starvation for 24 h. Meanwhile, the cells were treated with glutamine (Glu) starvation or α-Ketoglutarate (α-KG, 5 mM). The levels of α-KG (**B**) and succinate (Suc, **C**) were measured in each group. In addition, cell viability (**D**), MDA (**E**), 4-HNE (**F**) as well as the expression of *PTGS2* (G) were also measured respectively to evaluate ferroptosis in different groups. Results are expressed as mean ± SD, and the *P* value less than 0.05 was considered statistically significant. *:*P* < 0.05 compared with the Ctrl group in CC(+)Glu(+)α-KG(−) condition. #:*P* < 0.05 compared between Ctrl group and Overexpression (OE) group in same condition. &: *P* < 0.05 compared with the OE group in CC(+)Glu(+)α-KG (−) condition.

Both TCA cycle and glutaminolysis have been demonstrated to be necessary in cysteine deprivation-induced ferroptosis, and the block of TCA cycle or the absence of glutamine may relieve CC starvation or erastin-induced ferroptosis (Fig. 3A) [16]. To test the effect of COX7A1 on TCA cycle, the downstream metabolites of glutaminolysis were examined in our study. We found that the basal levels of α-Ketoglutarate (α-KG) and succinate (Suc) were higher in COX7A1 overexpression group than Ctrl group, and the absence of glutamine reduced the levels of α-KG and Suc effectively. The treatment with α-KG also increased the cellular Suc level, and the response in Overexpression group was more severe compared with Ctrl group (Fig. 3B, C), indicating that COX7A1 promotes mitochondrial TCA cycle in human NSCLC cells. In addition, the effect of glutamine starvation and α-KG treatment was also evaluated in CC starvation-induced ferroptosis model. The results showed that the ferroptosis was rescued significantly by glutamine starvation, but was aggravated by α-KG treatment, which also led to more obvious difference on ferroptosis between Ctrl group and Overexpression group (Fig. 3D–G). Thus, those results indicated that the effect of COX7A1 on mitochondrial TCA cycle contributed to the enhanced sensitivity to cysteine deprivation-induced ferroptosis in the cells with COX7A1 overexpression.

### COX7A1 increases the activity of Complex IV in mitochondrial ETC in NSCLC cells

**Fig. 4:**
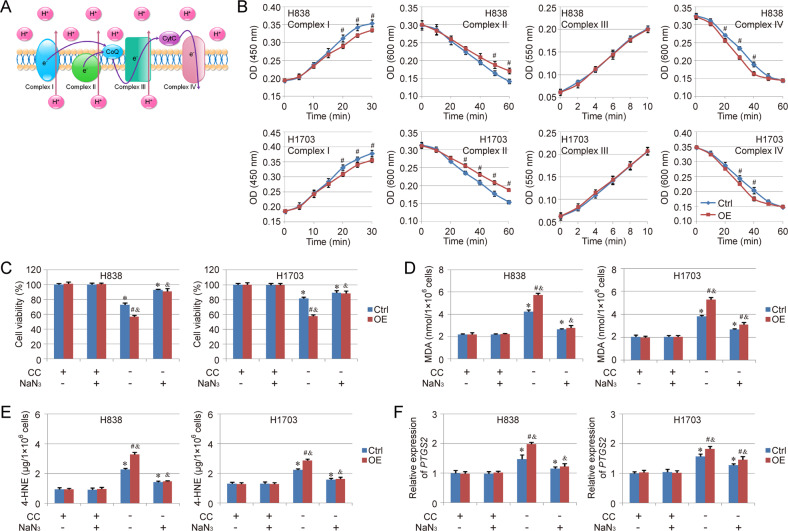
Effect of COX7A1 on mitochondrial ETC in NSCLC cells. The scheme of mitochondrial electron transport chain (ETC) complexes was showed in **A**. The activity of different complexes (Complex I to IV) in mitochondrial ETC were analyzed respectively (**B**). The ferroptosis model was induced by cellular cysteine (CC) starvation for 24 h. Meanwhile, the cells were treated with Complex IV inhibitor, NaN_3_ (15 mM). Then cell viability (**C**), MDA (**D**), 4-HNE (**E**) as well as the expression of *PTGS2* (**F**) were also measured respectively to evaluate ferroptosis in different groups. Results are expressed as mean ± SD, and the P value less than 0.05 was considered statistically significant. *:*P* < 0.05 compared with the Ctrl group in CC(+)NaN_3_(−) condition. #:*P* < 0.05 compared between Ctrl group and Overexpression (OE) group in same condition. &:*P* < 0.05 compared with the OE group in CC(+)NaN_3_(−) condition.

The mitochondrial ETC is a series of electron transporters, which are embedded in the mitochondrial inner membrane (Fig. 4A). Mitochondrial membrane potential is generated by ETC, which is necessary for ATP production. The different components (Complex I to IV) in ETC are also required for ferroptosis induced by cysteine deprivation. Therefore, the effect of COX7A1 on different ETC components was detected in NSCLC cells herein. Our results indicated that COX7A1 overexpression increased mitochondrial membrane potential and promoted ATP production in NSCLC cells (Figure S2A, B). Besides, the activity of Complex IV in mitochondrial ETC was enhanced by COX7A1 overexpression, while COX7A1 inhibited the activity of Complex I and Complex II, and no obvious difference could be found about the Complex III activity between Ctrl group and Overexpression group (Fig. 4B), suggesting that the enhanced activity of Complex IV could play an important role in the function of COX7A1 on ferroptosis regulation. Thus, NaN_3_, a inhibitor of Complex IV in mitochondrial ETC, was used to confirm the mechanism. We found that the treatment with NaN_3_ relieved cysteine deprivation-induced ferroptosis in both Ctrl group and Overexpression group. More importantly, after the treatment with NaN_3_, there were no obvious difference between Ctrl group and Overexpression group in most ferroptosis-related detections, except the MDA level and *PTGS2* expression in H1703 cells (Fig. 4C–F).

### COX7A1 blocks mitochondrial dynamics as well as mitochondrial biogenesis and mitophagy via inhibiting autophagic flux

Our previous study has indicated that COX7A1 could block the autophagic flux in NSCLC cells. Herein, the results further confirmed this conclusion. We found that the overexpression of COX7A1 led to the up-regulation of p62 and LC3 expression obviously (Figure S3A). Besides, the autophagic flux was analyzed by the transfection of tandem mRFP-GFP-LC3 reporter construct (yellow puncta: autophagosomes; red puncta: autolysosomes), and the results showed the increased amount of yellow puncta in Overexpression group compared with Ctrl group, indicating that COX7A1 overexpression resulted in the accumulation of autophagosomes. In addition, the treatment with rapamycin (Rap), an autophagy activator, rescued the block of autophagic flux, and promoted the formation of autolysosomes (red puncta) in both Ctrl group and Overexpression group (Figure S3B).

Autophagy is considered as a key determinant for mitochondrial metabolism and function. Even though COX7A1 blocked the autophagic flux in NSCLC cells, the content of mitochondria didn’t show obvious difference between Ctrl group and Overexpression group, which was indicated by the similar MitoTracker signaling and mitochondrial protein (TOM20 and TIM23) levels (Figure S4A, B). However, our previous results showed that COX7A1 inhibited the activity of Complex I and Complex II in mitochondrial ETC (Fig. 4B). Therefore, COX7A1 should also hold a negative regulatory function in mitochondrial metabolism and function.

**Fig. 5:**
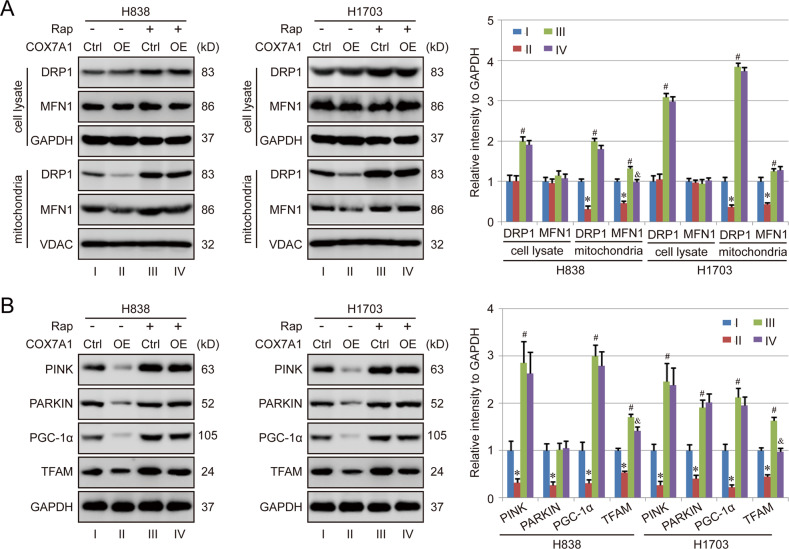
COX7A1 blocks mitochondrial dynamics as well as mitochondrial biogenesis and mitophagy via affecting autophagy. The NSCLC cells were treated with rapamycin (Rap, 100 nM) for 24 h, then harvested for western blot detection. Herein, mitochondrial fission-associated protein DRP1 and mitochondrial fusion-associated protein MFN1 were measured in total cell lysate and mitochondria respectively (**A**). Besides, mitophagy-associated proteins (PINK and PARKIN) as well as mitochondrial biogenesis-associated proteins (PGC-1α and TFAM) were also detected in whole cell lysate (**B**). Results are expressed as mean ± SD, and the *P* value less than 0.05 was considered statistically significant. *:*P* < 0.05 compared between Group I and Group II. #:*P* < 0.05 compared between Group I and Group III. &*:P* < 0.05 compared between Group III and Group IV.

Mitochondria are dynamic organelles, which engage in the coordinated cycles of fission and fusion. Thus, the mitochondrial dynamics as well as mitochondrial biogenesis and mitophagy were further evaluated in our following study. Herein, mitochondrial fission-associated protein DRP1 and mitochondrial fusion-associated protein MFN1 were measured respectively. The results showed that the levels of both DRP1 and MFN1 didn’t show significant difference in the total cell lysate from Ctrl goup and Overexpression group. However, COX7A1 downregulated the protein levels of DRP1 and MFN1 in mitochondria obviously, which could be abolished by Rap treatment (Fig. 5A). In addition, the overexpression of COX7A1 suppressed the expression of mitophagy-associated proteins (PINK and PARKIN) as well as mitochondrial biogenesis-associated proteins (PGC-1α and TFAM), and Rap treatment also rescued the inhibition effectively (Fig. 5B). Totally, those results revealed that COX7A1 could block mitochondrial dynamics as well as mitochondrial biogenesis and mitophagy via inhibiting autophagic flux, which could be rescued by autophagy activation.

### Rapamycin enhances the sensitivity of NSCLC cells to cysteine deprivation-induced ferroptosis via regulating TCA cycle and mitochondrial ETC

**Fig. 6:**
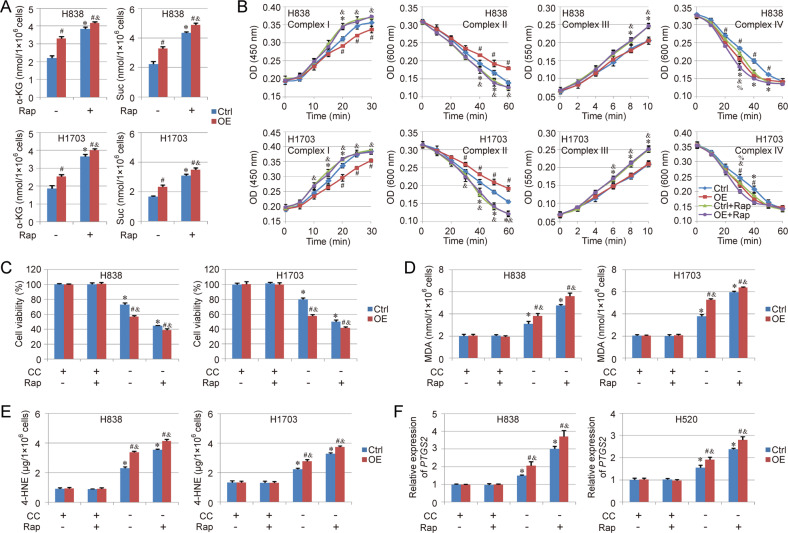
Rapamycin enhances the sensitivity of NSCLC cells to cysteine deprivation-induced ferroptosis through regulating TCA cycle and mitochondrial ETC. The NSCLC cells were treated with rapamycin (Rap, 100 nM) for 24 h, then the ferroptosis model was induced by cellular cysteine (CC) starvation for another 24 h.The levels of α-KG and Suc (**A**) as well as the activity of different complexes (Complex I to IV) in mitochondrial ETC (**B**) were analyzed respectively. In addition, cell viability (**C**), MDA (**D**), 4-HNE (**E**) as well as the expression of *PTGS2* (**F**) were also detected respectively to evaluate ferroptosis in different groups. Results are expressed as mean ± SD, and the P value less than 0.05 was considered statistically significant. In Fig. 2B, *:*P* < 0.05 compared between Ctrl group and Ctrl+Rap group. #:*P* < 0.05 compared between Ctrl group and Overexpression (OE) group; &:*P* < 0.05 compared between OE group and OE + Rap group; %:*P* < 0.05 compared between Ctrl+Rap group and OE + Rap group. In other figures, *:*P* < 0.05 compared with the Ctrl group in CC(+)Rap(−) condition. #:*P* < 0.05 compared between Ctrl group and Overexpression (OE) group in same condition. &:*P* < 0.05 compared with the OE group in CC(+)Rap(−) condition.

**Fig. 7:**
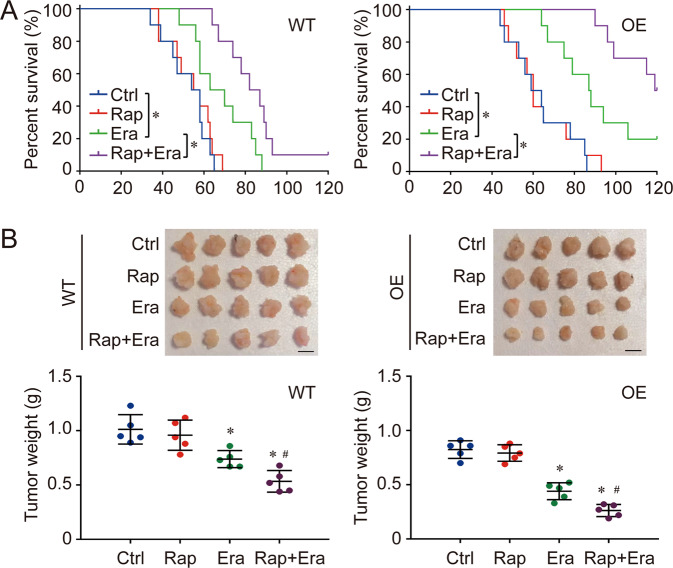
Anti-tumor efficacy of erastin (Era) and Rap in xenograft mouse model. WT and COX7A1 overexpressed (OE) H838 cells were injected into NOG mice, and the mice were further treated with Era and Rap. The survival rate (*n* = 10) within 120 days was also evaluated in different groups (A).The univariate cox proportional hazard regression assay was performed to compare the difference of survival rate between different group. *:*P* < 0.05 compared between two different groups. In addition, the mice were sacrificed and tumor weight (*n* = 5) was measured in each group (**B**, Scale bar = 1 cm). Results are expressed as mean ± SD, and the *P* value less than 0.05 was considered statistically significant. *:*P* < 0.05 compared with the Ctrl group. #:*P* < 0.05 compared between Era group and Rap + Era group.

As Rap enhanced the mitochondrial dynamics as well as mitochondrial biogenesis and mitophagy, the effect of Rap on TCA cycle and mitochondrial ETC was detected in this study. We found the basal levels of α-KG and Suc were increased significantly in both Ctrl goup and Overexpression group after the Rap treatment. Especially, the difference between Ctrl goup and Overexpression group was reduced by the treatment of Rap (Fig. 6A). The activity of different ETC components was also evaluated, and the results showed that the activity of all ETC components (Complex I to IV) was enhanced by Rap treatment. Beside Complex IV, no obvious difference could be found between Ctrl group and Overexpression group after the treatment of Rap (Fig. 6B). The response to cysteine deprivation-induced ferroptosis was further evaluated in NSCLC cells treated with Rap. The results indicated that Rap treatment increased the sensitivity of NSCLC cells to cysteine deprivation-induced ferroptosis in both Ctrl group and Overexpression group. Consistent with TCA cycle detection, Rap treatment decreased the difference on ferroptosis response between Ctrl group and Overexpression group, even though the ferroptosis level in COX7A1 overexpression group are still higher than Ctrl group (Fig. 6C–F). In addition, the therapeutic action of the combination of erastin (Era, ferroptosis inducer via cysteine deprivation) and Rap against NSCLC was also evaluated in vivo. Our results showed that the combination of Era and Rap hold better therapy function than single Era or Rap. In the Rap+Era group, the survival rate was improved and the tumor weight was lower compare with signal Era or Rap group (Fig. 7A, B).

## Discussion

**Fig. 8:**
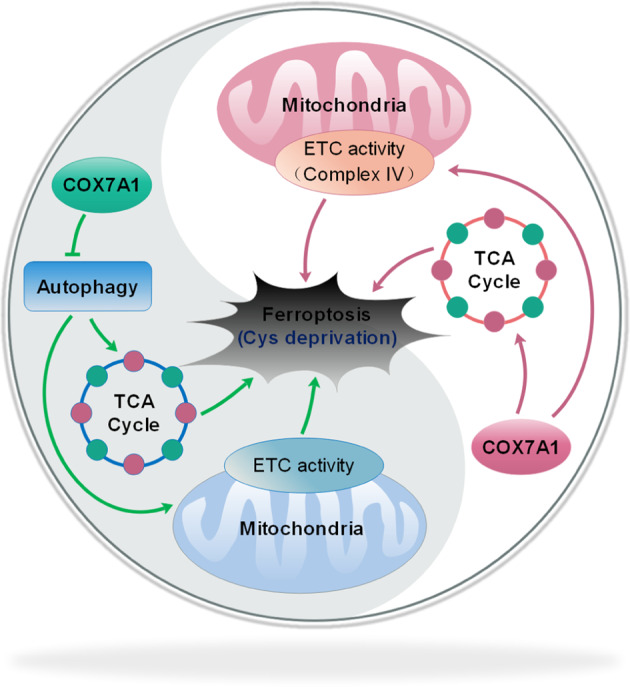
Proposed model for the relationship between COX7A1 and cysteine deprivation-induced ferroptosis. COX7A1 increased the sensitivity of NSCLC cells to the ferroptosis induced by cysteine (Cys) deprivation via enhancing TCA cycle and the activity of complex IV in mitochondrial ETC. Meanwhile, COX7A1 suppressed mitochondrial activity through blocking autophagy.

The functional relevance of mitochondria in ferroptosis has been investigated by some researchers in recent years. For example, both mitochondrial damage and mitochondrial ROS production are associated with ferroptosis [1], and some compound which targets mitochondria also can be used as ferroptosis inhibitor [37]. Moreover, the suppression of mitochondrial TCA cycle or ETC mitigates the accumulation of lipid ROS as well as ferroptosis [16]. Therefore, all of these studies indicate the potential involvement of mitochondria in the process of ferroptosis. However, some scientists also show opposite results against the central role of mitochondria in ferroptosis. Gaschler MM et al. explored the functional relevance of mitochondria to ferroptosis suppression induced by ferrostatins. Their results showed that endoplasmic reticulum might play a more important role in the ferroptosis inhibition, and mitochondria were not required for the ferroptosis regulation. Moreover, no significant correlation could be found between mitochondrial localization of ferrostatins and their anti-ferroptosis function [38]. Therefore, the accurate functional relevance of mitochondria in ferroptosis regulation could be different in different models, and still need our further investigation. In this study, we mainly explored the effect of COX7A1 on mitochondrial metabolism and cellular sensitivity to ferroptosis. On the one hand, our results showed that TCA cycle and the activity of complex IV in mitochondrial ETC could be enhanced by COX7A1 overexpression, which increased the sensitivity of NSCLC cells to the ferroptosis induced by cysteine deprivation. On the other hand, COX7A1 blocked autophagic flux and inhibited mitochondrial dynamics as well as mitochondrial biogenesis and mitophagy, which influence the activity of complex I and II in mitochondrial ETC. Moreover, Rap, an autophagy activator, could relieve the blockage induced by COX7A1 and further promoted the sensitivity to cysteine deprivation-induced ferroptosis in NSCLC cells (Fig. 8).

Autophagy is regarded as a natural survival-promoting pathway. In the process of autophagy, the unnecessary or dysfunctional components will be captured and degraded in lysosomes, and the useful components will be recycled to support the cellular metabolism. The autophagic elimination of mitochondria is called “mitophagy”, which is important to maintain the healthy state of mitochondria, as well as the balance between mitochondrial biogenesis and degradation. PINK1 is a kind of E3 ubiquitin ligase, and it also accumulates on the outer membrane of dysfunctional mitochondria and causes the binding between PARKIN and defective mitochondria, which further leads to the autophagic elimination of those mitochondria. In this study, we found that the overexpression of COX7A1 decreased the protein levels of both PINK1 and PARKIN, indicating the blocking effect of COX7A1 on mitophagy. In addition, our previous results have indicated the suppression of COX7A1 on PGC-1α (mitochondrial biogenesis activator) expression [25]. Herein, we also confirmed this conclusion, and found that another mitochondrial biogenesis regulator, TFAM, was downregulated by COX7A1 overexpression as well. Therefore, the phenomenon, no obvious change on the total amount of mitochondria, could be caused by the blockage in both mitochondrial biogenesis and elimination. Besides, we also notice the inhibition of mitochondrial fission and fusion were induced by COX7A1 overexpression, and both DRP1 (mitochondrial fission-associated protein) and MFN1 (mitochondrial fusion-associated protein) were downregulated in COX7A1 overexpression group compared with Ctrl group in mitochondria. The complex relationships among mitochondrial fission and fusion, as well as mitochondrial biogenesis and mitophagy, have gradually been revealed by many scientists in recent years. For example, mitochondria injury can be aggravated mitochondrial fission accompanied with mitophagy, resulting in the cell death through excessive self-consumption [39, 40]. Meanwhile, mitochondrial fission and fusion also can be eliminated by mitophagy in some degree [41]. Thus, which mechanism holds the major potential in the function of COX7A1 on mitochondrial activity and ferroptosis regulation, still needs our further investigation.

Rap is a specific inhibitor of mTOR signaling pathway. This compound has been demonstrated to hold effective therapeutic action against certain diseases, such as cancer [42, 43], diabetes [44, 45] and even some genetic disorders [46, 47]. In addition, Rap is also considered as a functional autophagy activator, which has been identified in various of cell lines. However, the detailed mechanism and the accurate downstream targets related with autophagy are still unclear for us [48–50]. Herein, our research indicated a novel function of Rap, enhancing the sensitivity of NSCLC cells to cysteine deprivation-induced ferroptosis. Some groups have showed that autophagy promotes TCA cycle via the regulation of amino acids metabolism [51], and Rap also can increase the activity of ETC complex [52]. Even though COX7A1 increased the activity of complex IV in mitochondrial ETC and enhanced TCA cycle, COX7A1 also inhibited mitochondrial dynamics as well as mitochondrial biogenesis and mitophagy via blocking autophagic flux, which might decrease the sensitivity of NSCLC cells to cysteine deprivation-induced ferroptosis. Meanwhile, Rep treatment relieved COX7A1-induced blockage of autophagy and obviously enhanced the sensitivity to cysteine deprivation-induced ferroptosis in NSCLC cells. Therefore, the combination of ferroptosis inducer and Rep could be a promising therapy mode against cancer in clinic.

The relevance between disease free survival rate and COX7A1 was evaluated in LUAD patients and LUSC patients using TCGA database. The high expression and low expression of COX7A1 were determined by the median value of COX7A1 mRNA transcripts per million (TPM). The univariate cox proportional hazard regression assay indicated that the mRNA level of COX7A1 wasn’t related with disease free survival rate in either LUAD patients or LUSC patients significantly (Figure S5A, B), even though the level of COX7A1 was significantly lower in the tumor tissues compared with normal lung tissues (Fig. 1A). It could be possible that COX7A1 holds more promising potential in the diagnosis, but not in the treatment of human NSCLC. However, ferroptosis inducers, such as erastin, haven’t been applied in the clinical treatment of NSCLC widely so far. Besides, the therapy modes for the patients in TCGA database are also different from each other. Thus, if the NSCLC patients are treated with ferroptosis inducers, whether the survival rate in high COX7A1 expression group could be higher than that in low COX7A1 expression group, still needs more clinical investigation.

In conclusion, our current study mainly addressed the link between COX7A1 and ferroptosis in human NSCLC model. COX7A1 increased the sensitivity of NSCLC cells to the ferroptosis induced by cysteine deprivation via promoting TCA cycle and the activity of complex IV in mitochondrial ETC. In addition, COX7A1 suppressed mitochondrial dynamics as well as mitochondrial biogenesis and mitophagy through blocking autophagic flux. Rap, an autophagy activator, relieved the blockage induced by COX7A1 and further strengthened the sensitivity to cysteine deprivation-induced ferroptosis. Although the crosstalk among COX7A1, mitochondrial metabolism, autophagy and ferroptosis needs to be investigated intensively, our research provides a novel insight into the therapy mode against human NSCLC.

## Supplementary information

Supplementary Figures

Reproducibility checklist

## Data Availability

All data generated in this study are included in the article and the additional files.

## References

[1] Dixon SJ, Lemberg KM, Lamprecht MR, Skouta R, Zaitsev EM, Gleason CE (2012). Ferroptosis: an iron-dependent form of nonapoptotic cell death. Cell.

[2] Stockwell BR, Friedmann Angeli JP, Bayir H, Bush AI, Conrad M, Dixon SJ (2017). Ferroptosis: A Regulated Cell Death Nexus Linking Metabolism, Redox Biology, and Disease. Cell.

[3] Bersuker K, Hendricks JM, Li Z, Magtanong L, Ford B, Tang PH (2019). The CoQ oxidoreductase FSP1 acts parallel to GPX4 to inhibit ferroptosis. Nature.

[4] Doll S, Freitas FP, Shah R, Aldrovandi M, da Silva MC, Ingold I (2019). FSP1 is a glutathione-independent ferroptosis suppressor. Nature.

[5] Lei P, Bai T, Sun Y (2019). Mechanisms of Ferroptosis and Relations With Regulated Cell Death: A Review. Front Physiol.

[6] Mao C, Liu XG, Zhang YL, Lei G, Yan YL, Lee H (2021). DHODH-mediated ferroptosis defence is a targetable vulnerability in cancer. Nature.

[7] Kenny EM, Fidan E, Yang Q, Anthonymuthu TS, New LA, Meyer EA (2019). Ferroptosis Contributes to Neuronal Death and Functional Outcome After Traumatic Brain Injury. Crit Care Med.

[8] Li Q, Han X, Lan X, Gao Y, Wan J, Durham F (2017). Inhibition of neuronal ferroptosis protects hemorrhagic brain. JCI Insight.

[9] Guan X, Li X, Yang X, Yan J, Shi P, Ba L (2019). The neuroprotective effects of carvacrol on ischemia/reperfusion-induced hippocampal neuronal impairment by ferroptosis mitigation. Life Sci.

[10] Liu P, Feng Y, Li H, Chen X, Wang G, Xu S (2020). Ferrostatin-1 alleviates lipopolysaccharide-induced acute lung injury via inhibiting ferroptosis. Cell Mol Biol Lett.

[11] Masaldan S, Belaidi AA, Ayton S, Bush AI (2019). Cellular Senescence and Iron Dyshomeostasis in Alzheimer’s Disease. Pharm (Basel).

[12] Xu T, Ding W, Ji X, Ao X, Liu Y, Yu W (2019). Molecular mechanisms of ferroptosis and its role in cancer therapy. J Cell Mol Med.

[13] Shi ZZ, Fan ZW, Chen YX, Xie XF, Jiang W, Wang WJ (2019). Ferroptosis in Carcinoma: Regulatory Mechanisms and New Method for Cancer Therapy. Onco Targets Ther.

[14] Friedmann Angeli JP, Krysko DV, Conrad M (2019). Ferroptosis at the crossroads of cancer-acquired drug resistance and immune evasion. Nat Rev Cancer.

[15] Li K, Lin C, Li M, Xu K, He Y, Mao Y (2022). Multienzyme-like Reactivity Cooperatively Impairs Glutathione Peroxidase 4 and Ferroptosis Suppressor Protein 1 Pathways in Triple-Negative Breast Cancer for Sensitized Ferroptosis Therapy. ACS Nano.

[16] Gao M, Yi J, Zhu J, Minikes AM, Monian P, Thompson CB (2019). Role of Mitochondria in Ferroptosis. Mol Cell.

[17] Burke PJ (2017). Mitochondria, Bioenergetics and Apoptosis in Cancer. Trends Cancer.

[18] Ying Z, Xiang G, Zheng L, Tang H, Duan L, Lin X (2019). Short-Term Mitochondrial Permeability Transition Pore Opening Modulates Histone Lysine Methylation at the Early Phase of Somatic Cell Reprogramming. Cell Metab.

[19] Du J, Zhou Y, Li Y, Xia J, Chen Y, Chen S (2020). Identification of Frataxin as a regulator of ferroptosis. Redox Biol.

[20] Liu J, Kuang F, Kroemer G, Klionsky DJ, Kang R, Tang D (2020). Autophagy-Dependent Ferroptosis: Machinery and Regulation. Cell Chem Biol.

[21] Little AG, Lau G, Mathers KE, Leary SC, Moyes CD (2018). Comparative biochemistry of cytochrome c oxidase in animals. Comp Biochem Physiol B Biochem Mol Biol.

[22] Kadenbach B, Huttemann M (2015). The subunit composition and function of mammalian cytochrome c oxidase. Mitochondrion.

[23] Arnaudo E, Hirano M, Seelan RS, Milatovich A, Hsieh CL, Fabrizi GM (1992). Tissue-specific expression and chromosome assignment of genes specifying two isoforms of subunit VIIa of human cytochrome c oxidase. Gene.

[24] West MD, Labat I, Sternberg H, Larocca D, Nasonkin I, Chapman KB (2018). Use of deep neural network ensembles to identify embryonic-fetal transition markers: repression of COX7A1 in embryonic and cancer cells. Oncotarget.

[25] Zhao L, Chen X, Feng Y, Wang G, Nawaz I, Hu L (2019). COX7A1 suppresses the viability of human non-small cell lung cancer cells via regulating autophagy. Cancer Med.

[26] Levy JMM, Towers CG, Thorburn A (2017). Targeting autophagy in cancer. Nat Rev Cancer.

[27] Rodolfo C, Di Bartolomeo S, Cecconi F (2016). Autophagy in stem and progenitor cells. Cell Mol Life Sci.

[28] Yoo SM, Jung YK (2018). A Molecular Approach to Mitophagy and Mitochondrial Dynamics. Mol Cells.

[29] Si L, Fu J, Liu W, Hayashi T, Mizuno K, Hattori S, et al. Silibinin-induced mitochondria fission leads to mitophagy, which attenuates silibinin-induced apoptosis in MCF-7 and MDA-MB-231 cells. Arch Biochem Biophys. 2020:108284. 10.1016/j.abb.2020.108284 32014401

[30] Zhou Y, Long Q, Wu H, Li W, Qi J, Wu Y (2020). Topology-dependent, bifurcated mitochondrial quality control under starvation. Autophagy.

[31] Liu P, Feng Y, Chen X, Wang G, Nawaz I, Hu L (2019). Paracrine action of human placental trophoblast cells attenuates cisplatin-induced acute kidney injury. Life Sci.

[32] Liu P, Tian W, Tao S, Tillotson J, Wijeratne EMK, Gunatilaka AAL (2019). Non-covalent NRF2 Activation Confers Greater Cellular Protection than Covalent Activation. Cell Chem Biol.

[33] Liu P, Rojo de la Vega M, Sammani S, Mascarenhas JB, Kerins M, Dodson M (2018). RPA1 binding to NRF2 switches ARE-dependent transcriptional activation to ARE-NRE-dependent repression. Proc Natl Acad Sci USA.

[34] Tao S, Liu P, Luo G, Rojo de la Vega M, Chen H, Wu T (2017). p97 Negatively Regulates NRF2 by Extracting Ubiquitylated NRF2 from the KEAP1-CUL3 E3 Complex. Mol Cell Biol.

[35] Zhao L, Feng Y, Chen X, Yuan J, Liu X, Chen Y (2016). Effects of IGF-1 on neural differentiation of human umbilical cord derived mesenchymal stem cells. Life Sci.

[36] Liu P, de la Vega MR, Dodson M, Yue F, Shi B, Fang D (2019). Spermidine Confers Liver Protection by Enhancing NRF2 Signaling Through a MAP1S-Mediated Noncanonical Mechanism. Hepatology.

[37] Krainz T, Gaschler MM, Lim C, Sacher JR, Stockwell BR, Wipf P (2016). A Mitochondrial-Targeted Nitroxide Is a Potent Inhibitor of Ferroptosis. ACS Cent Sci.

[38] Gaschler MM, Hu F, Feng H, Linkermann A, Min W, Stockwell BR (2018). Determination of the Subcellular Localization and Mechanism of Action of Ferrostatins in Suppressing Ferroptosis. ACS Chem Biol.

[39] Youle RJ, van der Bliek AM (2012). Mitochondrial fission, fusion, and stress. Science.

[40] Babbar M, Sheikh MS (2013). Metabolic Stress and Disorders Related to Alterations in Mitochondrial Fission or Fusion. Mol Cell Pharm.

[41] Twig G, Elorza A, Molina AJ, Mohamed H, Wikstrom JD, Walzer G (2008). Fission and selective fusion govern mitochondrial segregation and elimination by autophagy. EMBO J.

[42] Imrali A, Mao X, Yeste-Velasco M, Shamash J, Lu Y (2016). Rapamycin inhibits prostate cancer cell growth through cyclin D1 and enhances the cytotoxic efficacy of cisplatin. Am J Cancer Res.

[43] Liu Y, Pandeswara S, Dao V, Padron A, Drerup JM, Lao S (2017). Biphasic Rapamycin Effects in Lymphoma and Carcinoma Treatment. Cancer Res.

[44] Blagosklonny MV (2019). Fasting and rapamycin: diabetes versus benevolent glucose intolerance. Cell Death Dis.

[45] Barlow AD, Nicholson ML, Herbert TP (2013). Evidence for rapamycin toxicity in pancreatic beta-cells and a review of the underlying molecular mechanisms. Diabetes.

[46] Li J, Kim SG, Blenis J (2014). Rapamycin: one drug, many effects. Cell Metab.

[47] Koenig MK, Bell CS, Hebert AA, Roberson J, Samuels JA, Slopis JM (2018). Efficacy and Safety of Topical Rapamycin in Patients With Facial Angiofibromas Secondary to Tuberous Sclerosis Complex: The TREATMENT Randomized Clinical Trial. JAMA Dermatol.

[48] Sarkar S, Ravikumar B, Floto RA, Rubinsztein DC (2009). Rapamycin and mTOR-independent autophagy inducers ameliorate toxicity of polyglutamine-expanded huntingtin and related proteinopathies. Cell Death Differ.

[49] Russo R, Varano GP, Adornetto A, Nazio F, Tettamanti G, Girardello R (2018). Rapamycin and fasting sustain autophagy response activated by ischemia/reperfusion injury and promote retinal ganglion cell survival. Cell Death Dis.

[50] Liu Y, Yang F, Zou S, Qu L (2018). Rapamycin: A Bacteria-Derived Immunosuppressant That Has Anti-atherosclerotic Effects and Its Clinical Application. Front Pharm.

[51] Guo JY, Teng X, Laddha SV, Ma S, Van Nostrand SC, Yang Y (2016). Autophagy provides metabolic substrates to maintain energy charge and nucleotide pools in Ras-driven lung cancer cells. Genes Dev.

[52] Quarles E, Basisty N, Chiao YA, Merrihew G, Gu H, Sweetwyne MT (2020). Rapamycin persistently improves cardiac function in aged, male and female mice, even following cessation of treatment. Aging Cell.

