# Supplementary material for: COX7A1 enhances the sensitivity of human NSCLC cells to cystine deprivation-induced ferroptosis via regulating mitochondrial metabolism
Source: Cell Death Dis. 2022 Nov 23;13(11):988. doi: 10.1038/s41419-022-05430-3 (PMC9684511; doi:10.1038/s41419-022-05430-3)

Supplementary figure legends

Figure S1. Evaluation of COX7A1 overexpression in NSCLC cells. After lentivirus transduction, the stable positive cells were selected by blasticidin treatment (5 μg/ml) for 2 weeks. Then the cell were harvested for qPCR (A) and western blot detection (B). Then, the proliferation ability of COX7A1-overexpressed cell was evaluated using CCK-8 method (C). To analyze the ferroptosis level, MDA level was measured in each group (D). Results are expressed as mean±SD, and the P value less than 0.05 was considered statistically significant. *: P<0.05 compared between Ctrl group and Overexpression (OE) group.

Figure S2. Effect of COX7A1 on mitochondrial membrane potential (MMP) and ATP production. A. Evaluation of MMP via TMRE staining. B. Effect of COX7A1 on ATP production. Results are expressed as mean±SD, and the P value less than 0.05 was considered statistically significant. *: P<0.05 compared between Ctrl group and Overexpression (OE) group.

Figure S3. COX7A1 blocks autophagic flux in NSCLC cells. A. Effect of COX7A1 on p62 and LC3 expression. Results are expressed as mean±SD, and the P value less than 0.05 was considered statistically significant. *: P<0.05 compared between Ctrl group and Overexpression (OE) group. B. The autophagic flux was analyzed using mRFP-GFP-LC3 reporter. After transfected with mRFP-GFP-LC3 for 24 h, the cells were treated with rapamycin (Rap, 100 nM) for 24 h, and the fluorescence image cells was taken in different groups. Scale bar=10 μm.

Figure S4. Effect of COX7A1 on mitochondrial presence in NSCLC cells. A. the appearance of mitochondria was evaluated using MitoTracker® Green staining. B. Detection of mitochondrial proteins TOM20 and TIM23 with western blot. Results are expressed as mean±SD, and the P value less than 0.05 was considered statistically significant. *: P<0.05 compared between Ctrl group and Overexpression (OE) group.

Figure S5. Kaplan–Meier curves estimating the disease-free survival according to the mRNA expression levels of COX7A1 in LUAD patients (A) and LUSC patients (B) using TCGA database. The high expression and low expression of COX7A1 were determined by the median value of COX7A1 mRNA transcripts per million (TPM). The univariate cox proportional hazard regression assay was performed to analyze the relationship.

Figure S1

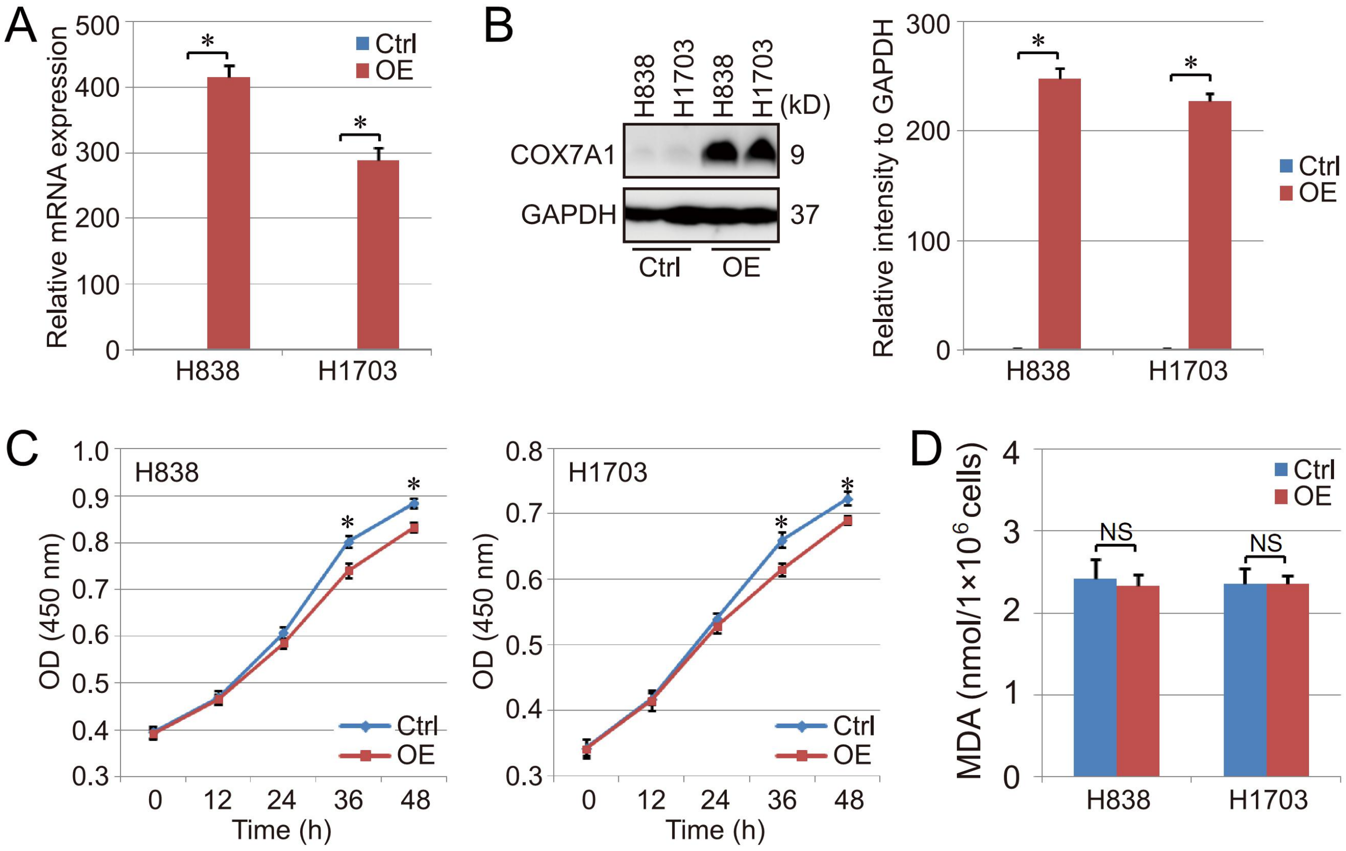

A
Relative mRNA expression
500
400
300
200
100
0
H838
H1703
Ctrl
OE
*
B
H838
H1703
H838
H1703
(kD)
COX7A1
9
GAPDH
37
Ctrl
OE
Relative intensity to GAPDH
300
200
100
0
C
H838
OD (450 nm)
1.0
0.9
0.8
0.7
0.6
0.5
0.4
0.3
0
12
24
36
48
Time (h)
H1703
D
MDA (nmol/1×10^6 cells)
4
3
2
1
0
NS

Figure S2

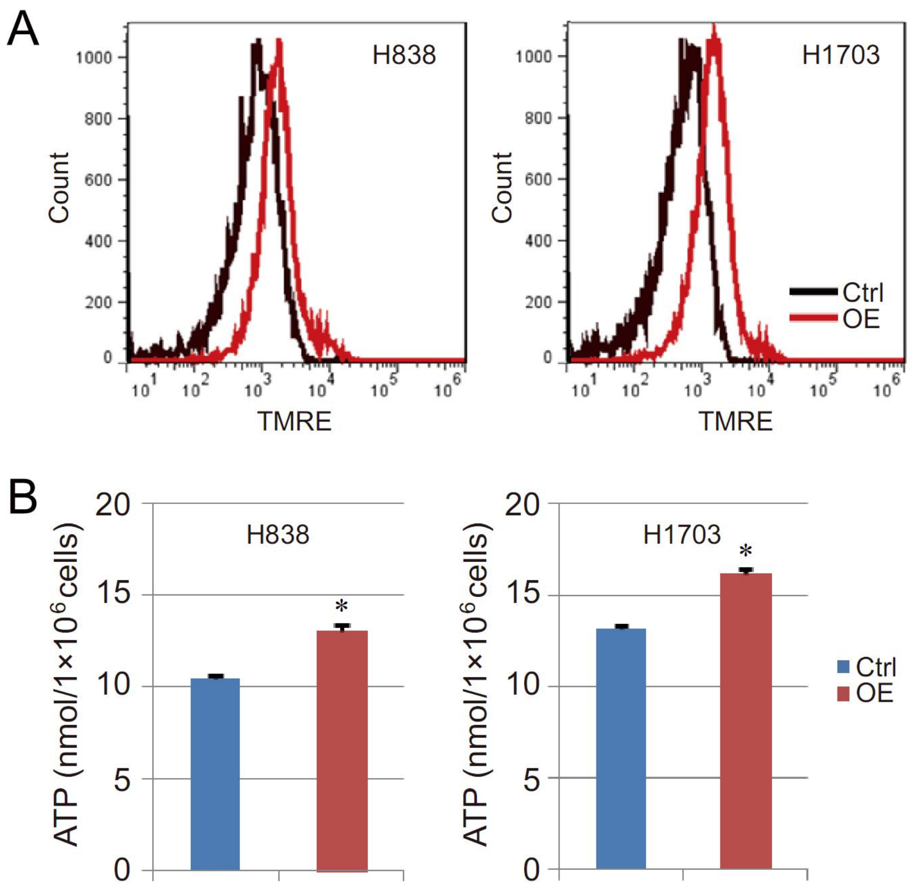
A
H838
H1703
Count
1000
800
600
400
200
0
TMRE
Ctrl
OE
B
ATP (nmol/1×10⁶cells)
20
15
10
5
0
*

Figure S3

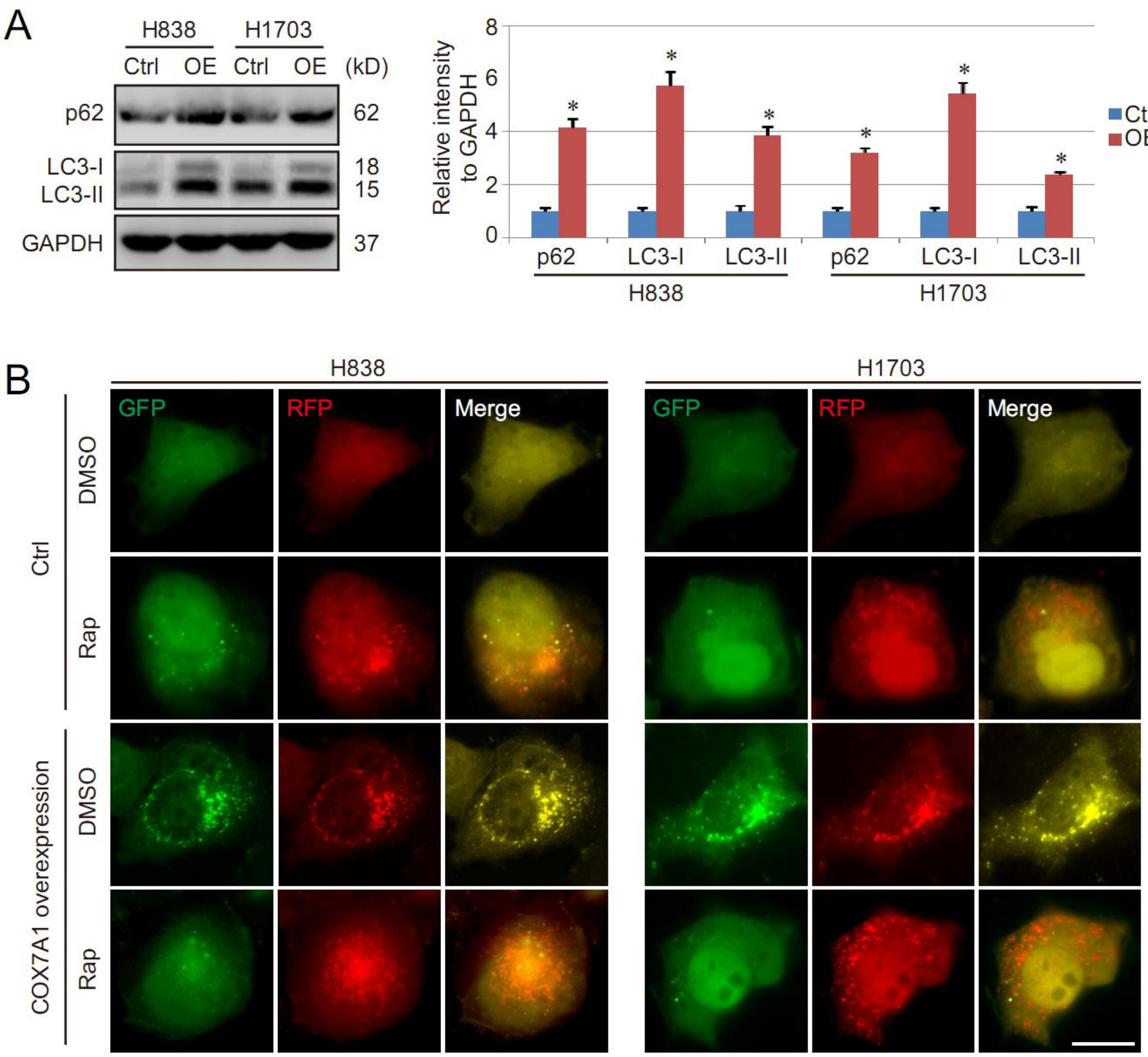
A
H838
H1703
Ctrl
OE
Ctrl
OE
(kD)
p62
62
LC3-I
LC3-II
18
15
GAPDH
37
Relative intensity to GAPDH
8
6
4
2
0
*
Ctrl
OE
p62
LC3-I
LC3-II
p62
LC3-I
LC3-II
H838
H1703
B
H838
H1703
GFP
RFP
Merge
GFP
RFP
Merge
DMSO
Rap
Ctrl
DMSO
Rap
COX7A1 overexpression

Figure S4

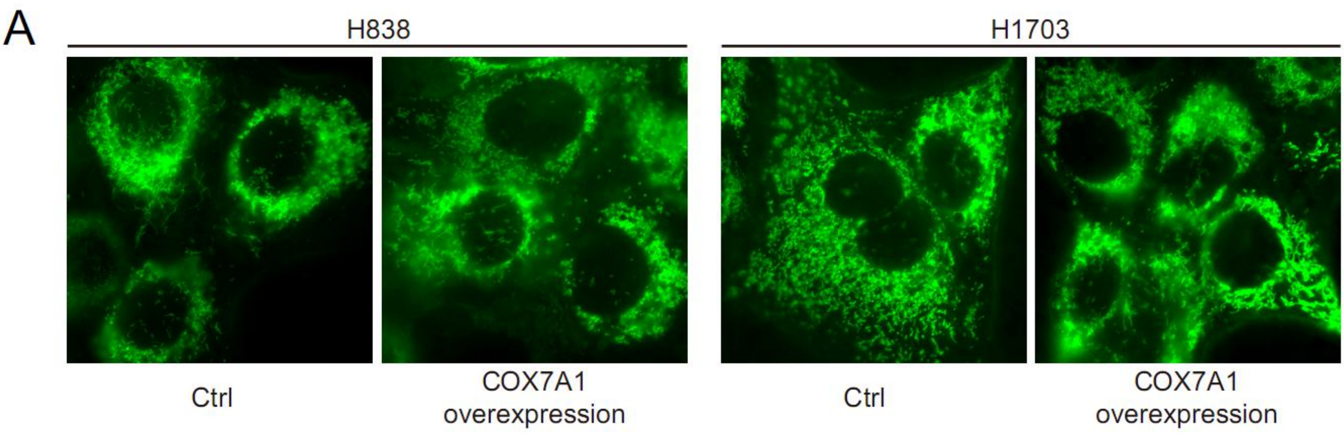
A
H838
H1703
Ctrl
COX7A1 overexpression
Ctrl
COX7A1 overexpression

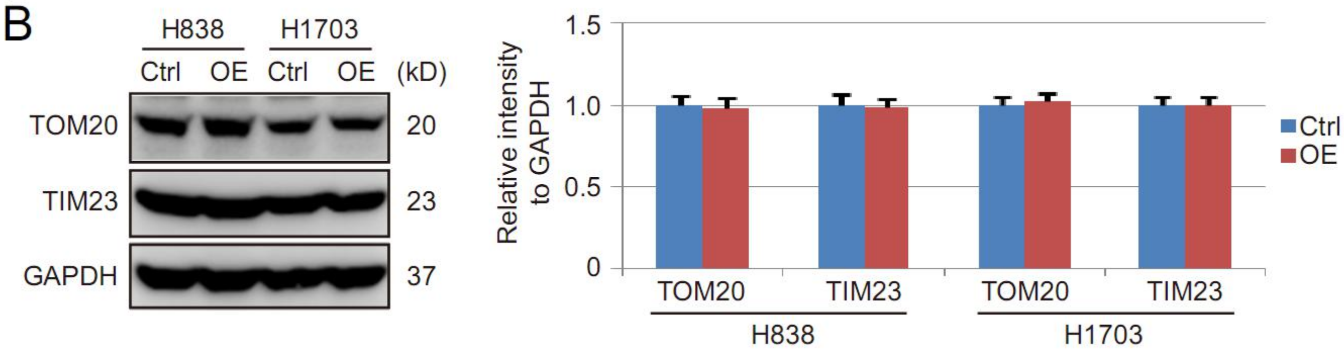
B
H838
H1703
Ctrl
OE
Ctrl
OE
(kD)
TOM20
20
TIM23
23
GAPDH
37
Relative intensity to GAPDH
1.5
1.0
0.5
0
TOM20
TIM23
TOM20
TIM23
H838
H1703
Ctrl
OE

Figure S5

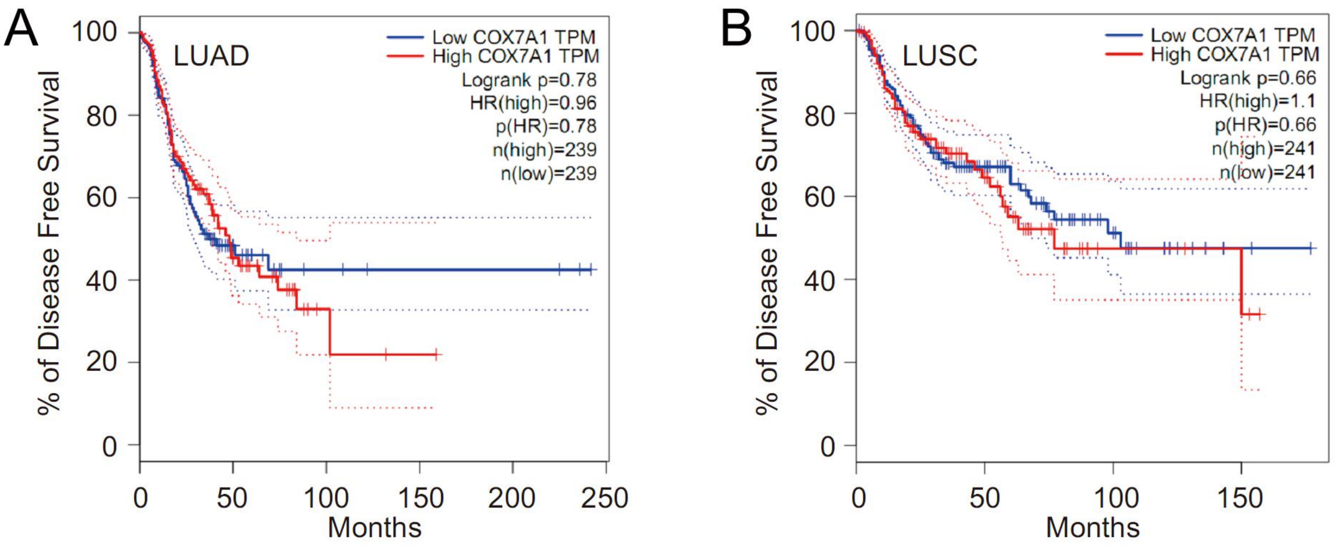
A
LUAD
Low COX7A1 TPM
High COX7A1 TPM
Logrank p=0.78
HR(high)=0.96
p(HR)=0.78
n(high)=239
n(low)=239
% of Disease Free Survival
Months
B
LUSC
Low COX7A1 TPM
High COX7A1 TPM
Logrank p=0.66
HR(high)=1.1
p(HR)=0.66
n(high)=241
n(low)=241
% of Disease Free Survival
Months

Original western blot images

Figure 1C-COX7A1

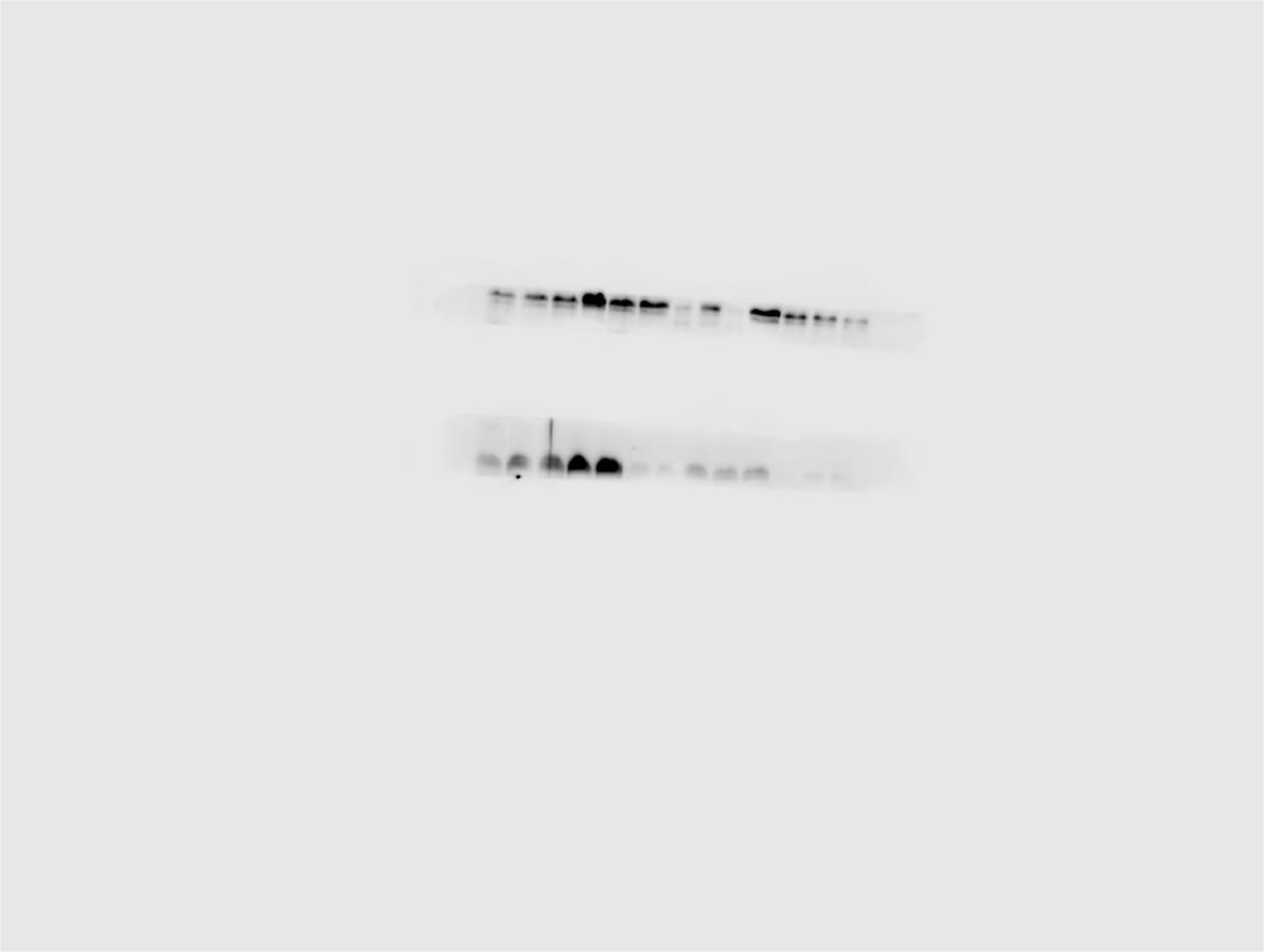

Figure 1C-GAPDH

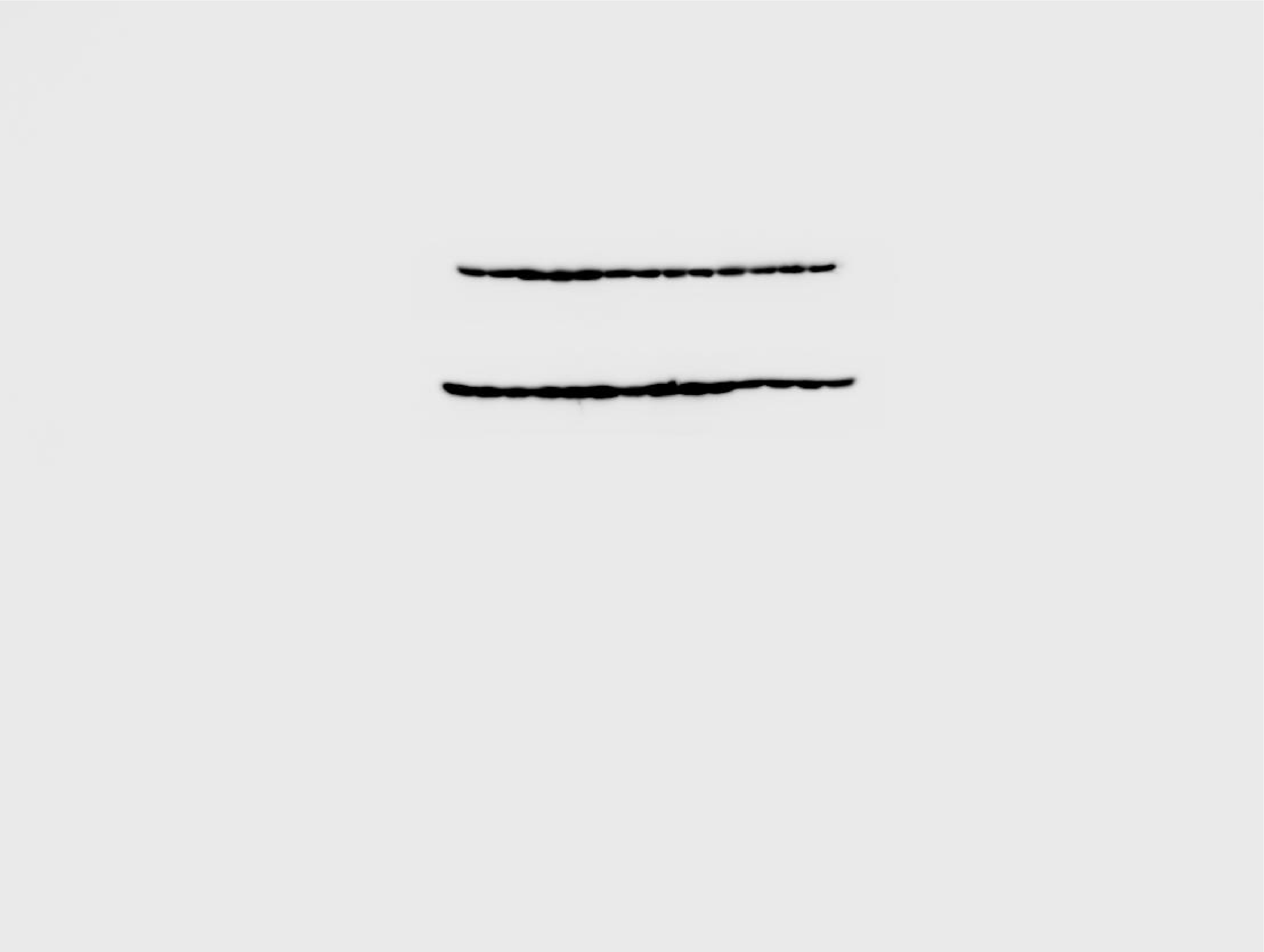

Figure 5A-H838 DRP1

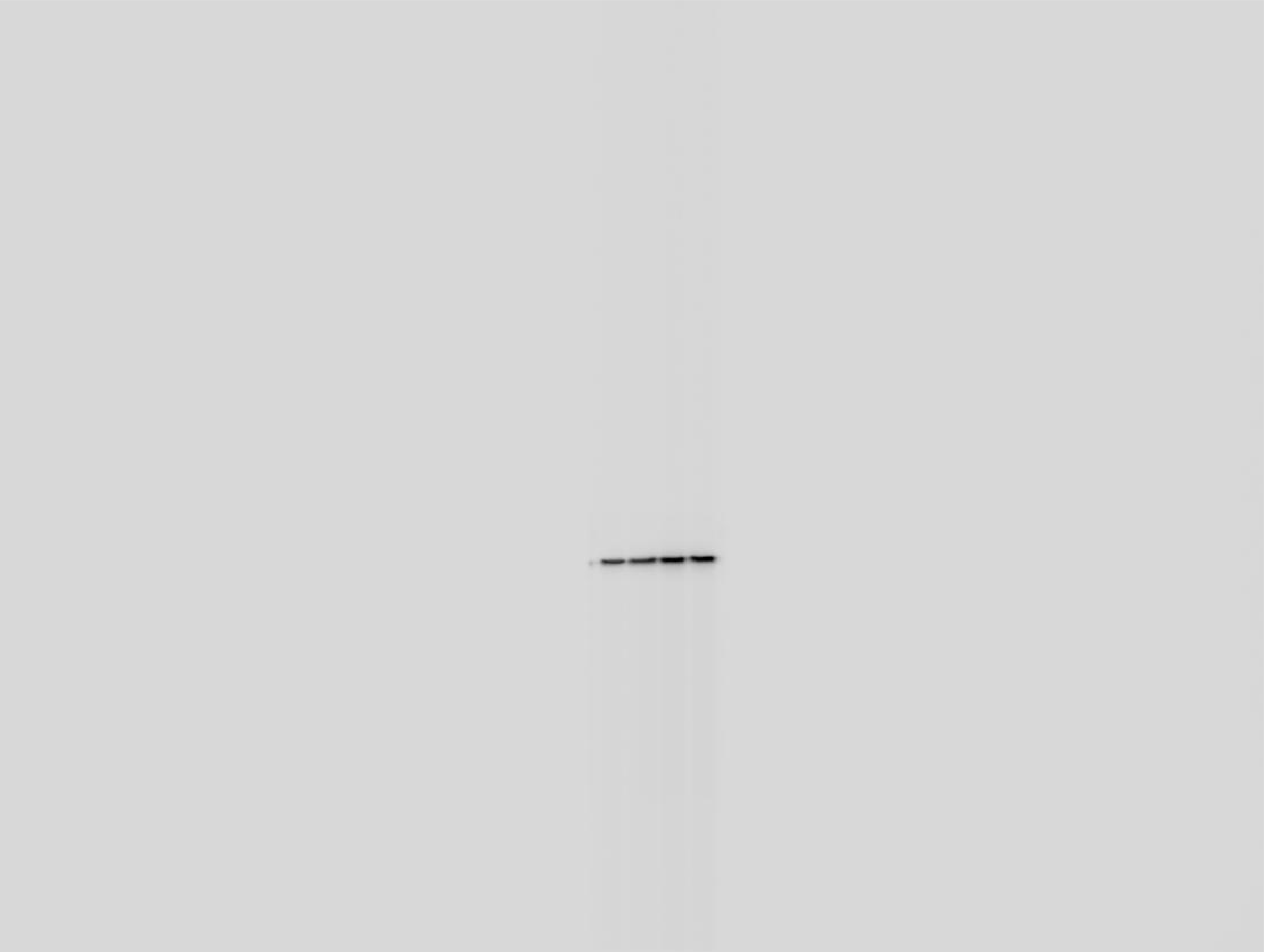

Figure 5A-H838 MFN1

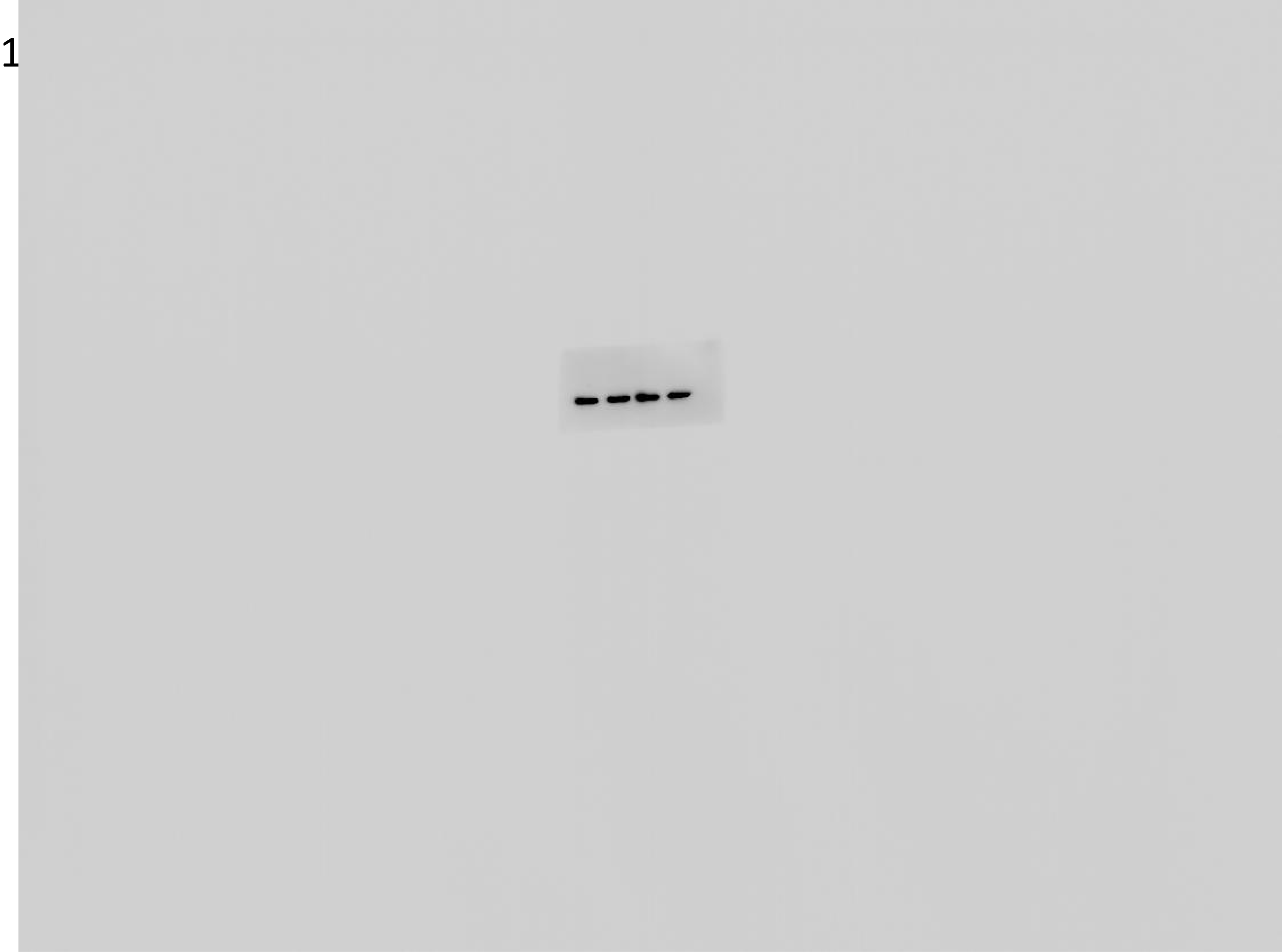

Figure 5A-H838 GAPDH

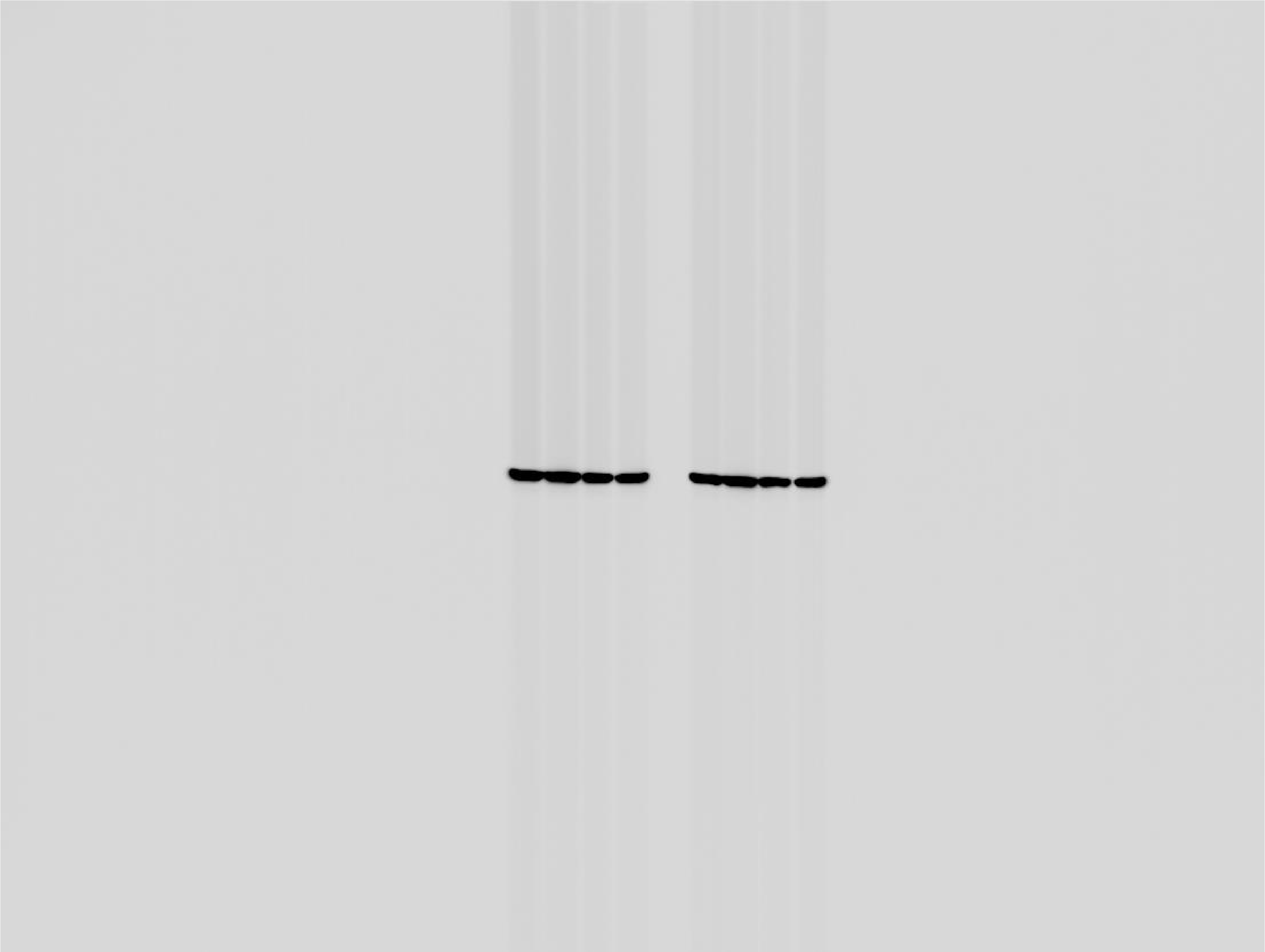

Figure 5A-H838
mito DRP1

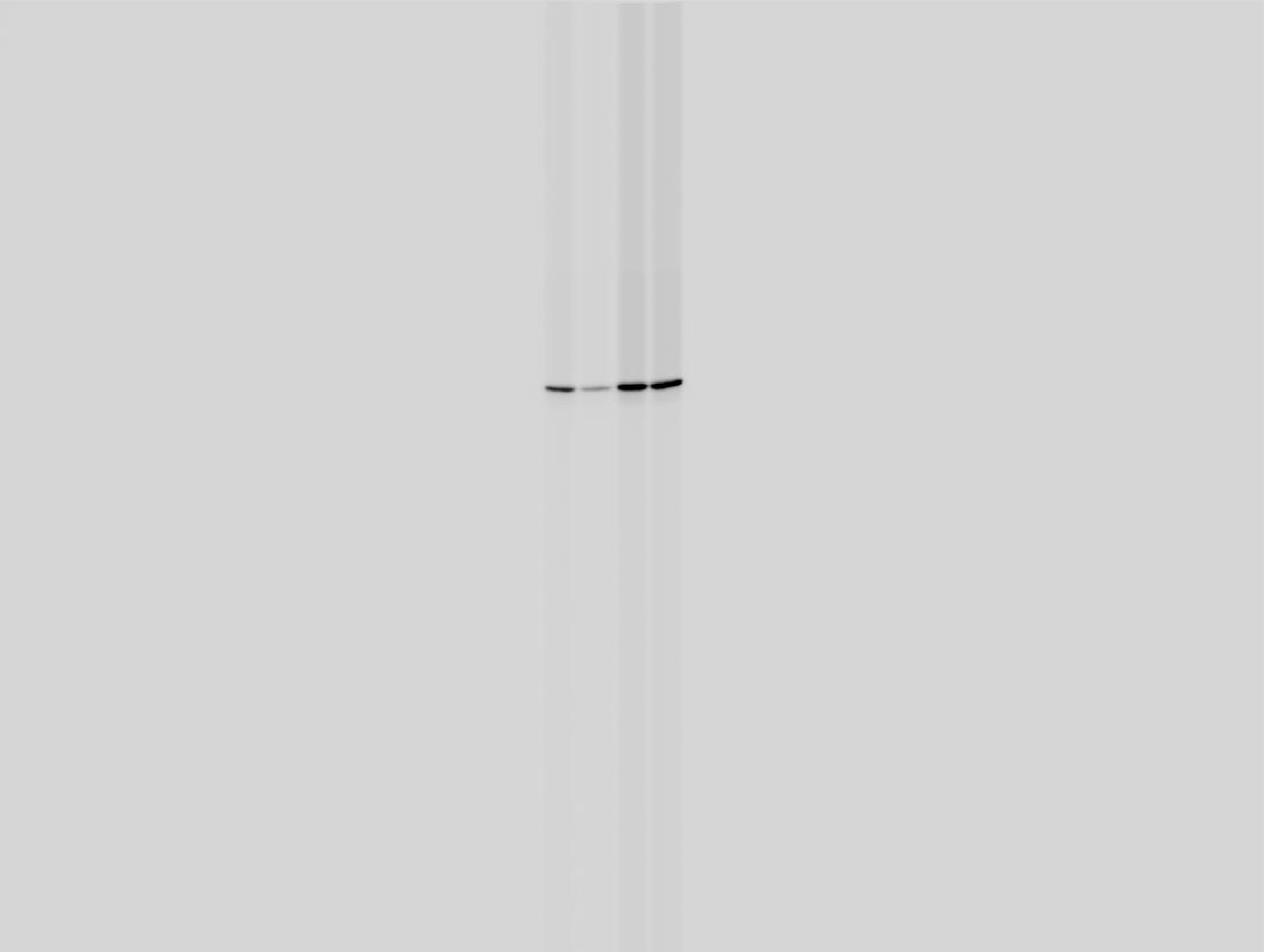

Figure 5A-H838
mito MFN1

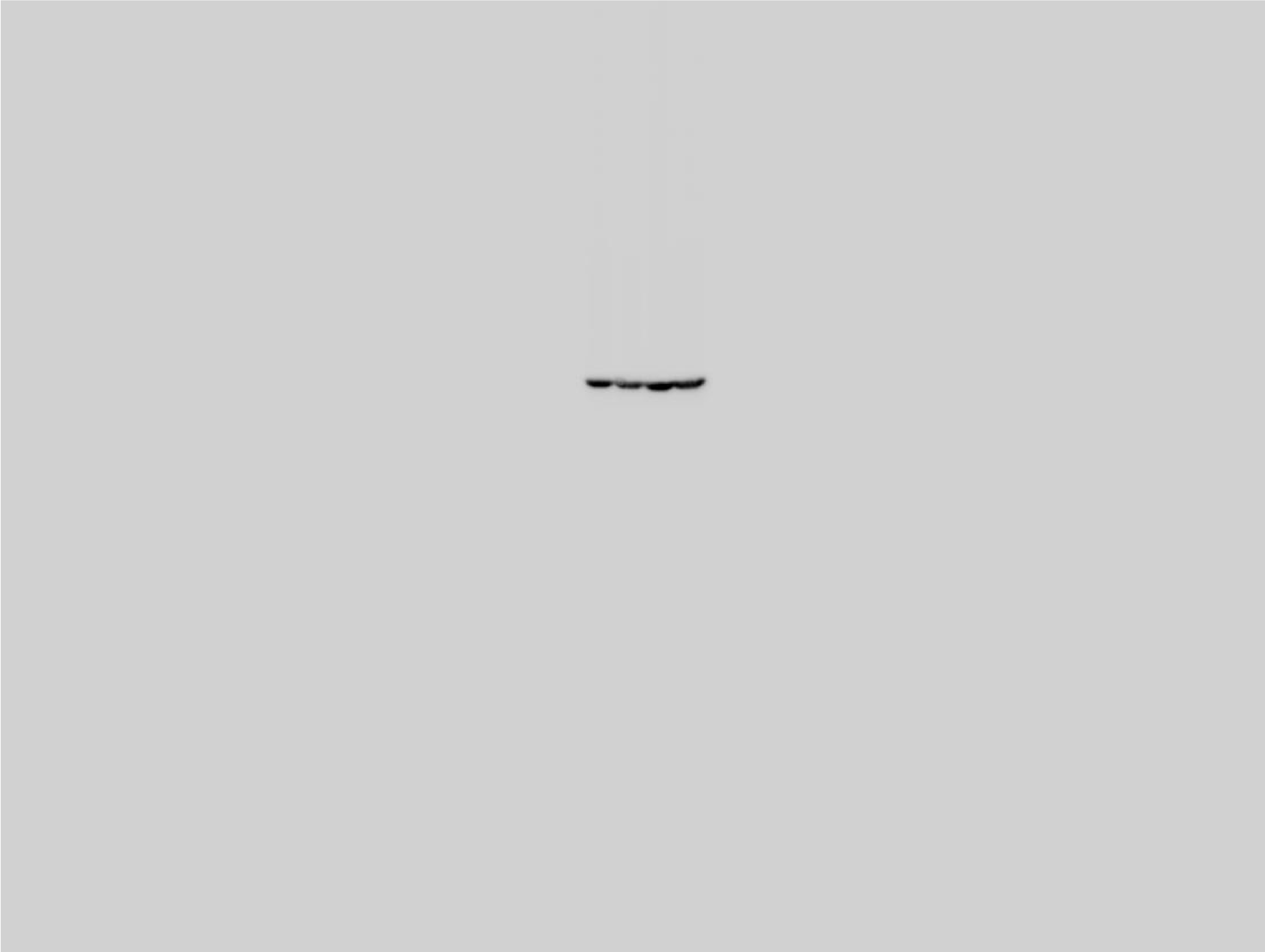

Figure 5A-H838

VDAC

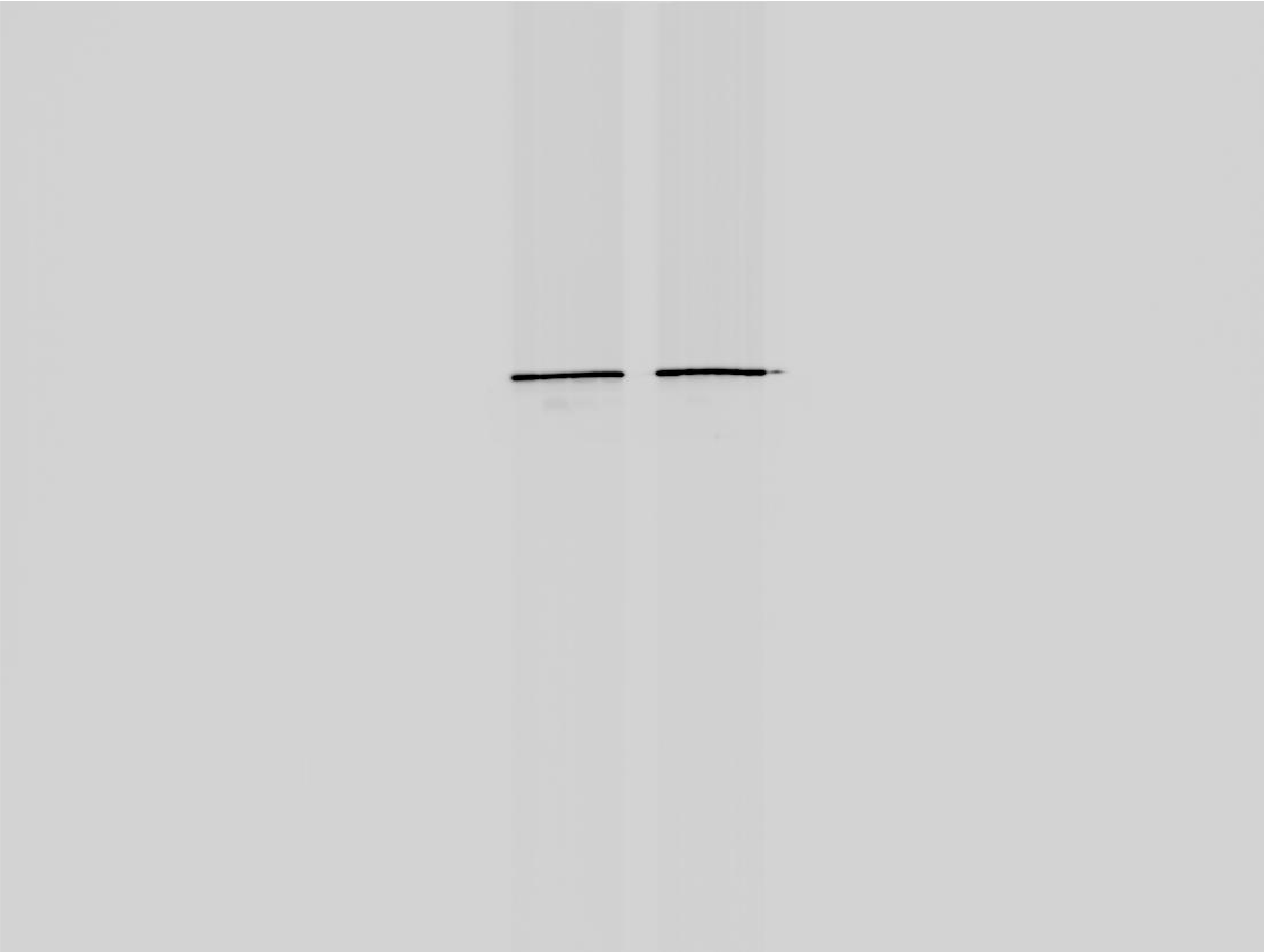

Figure 5A-H1703 DRP1

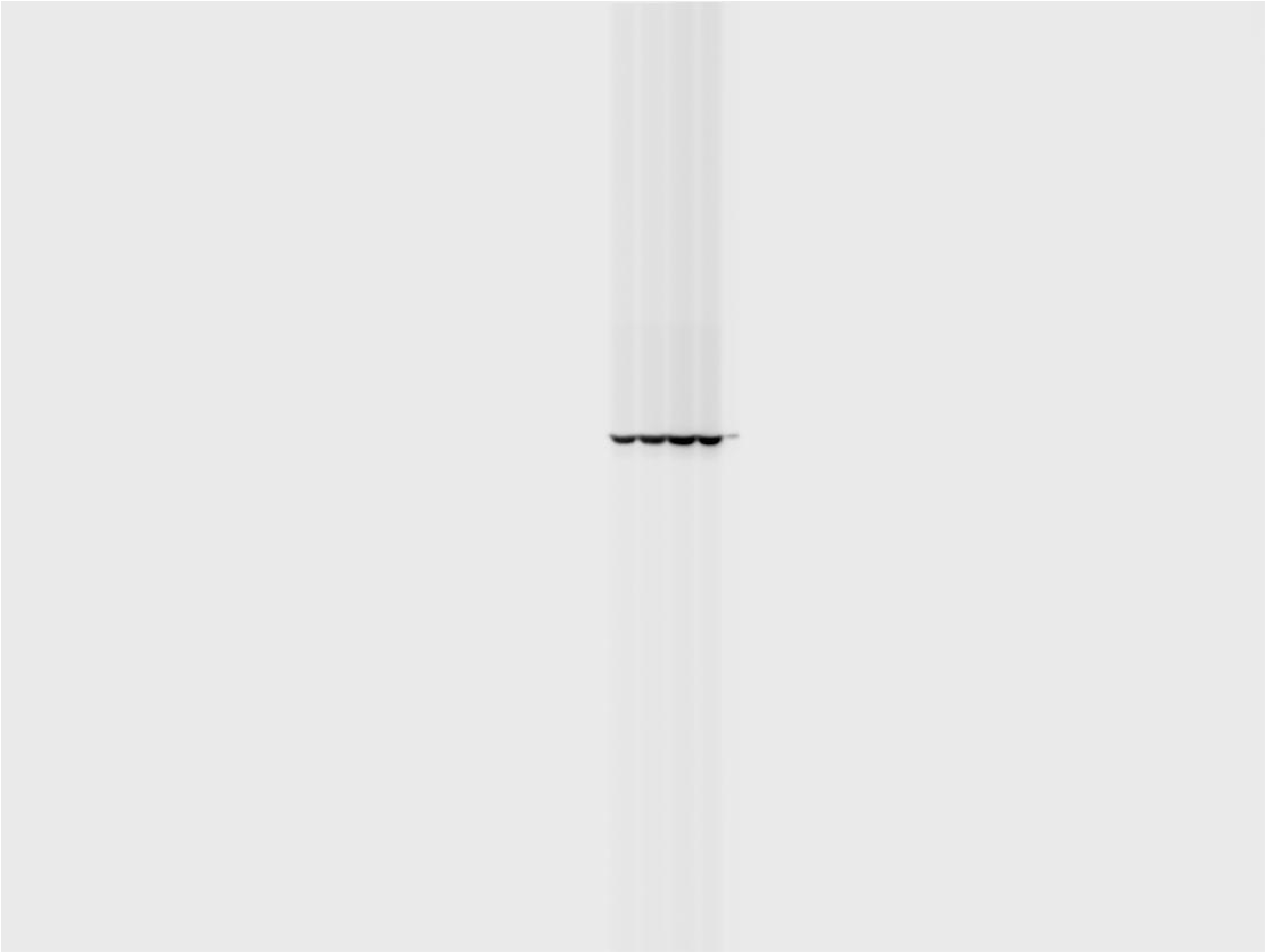

Figure 5A-H1703
MFN1

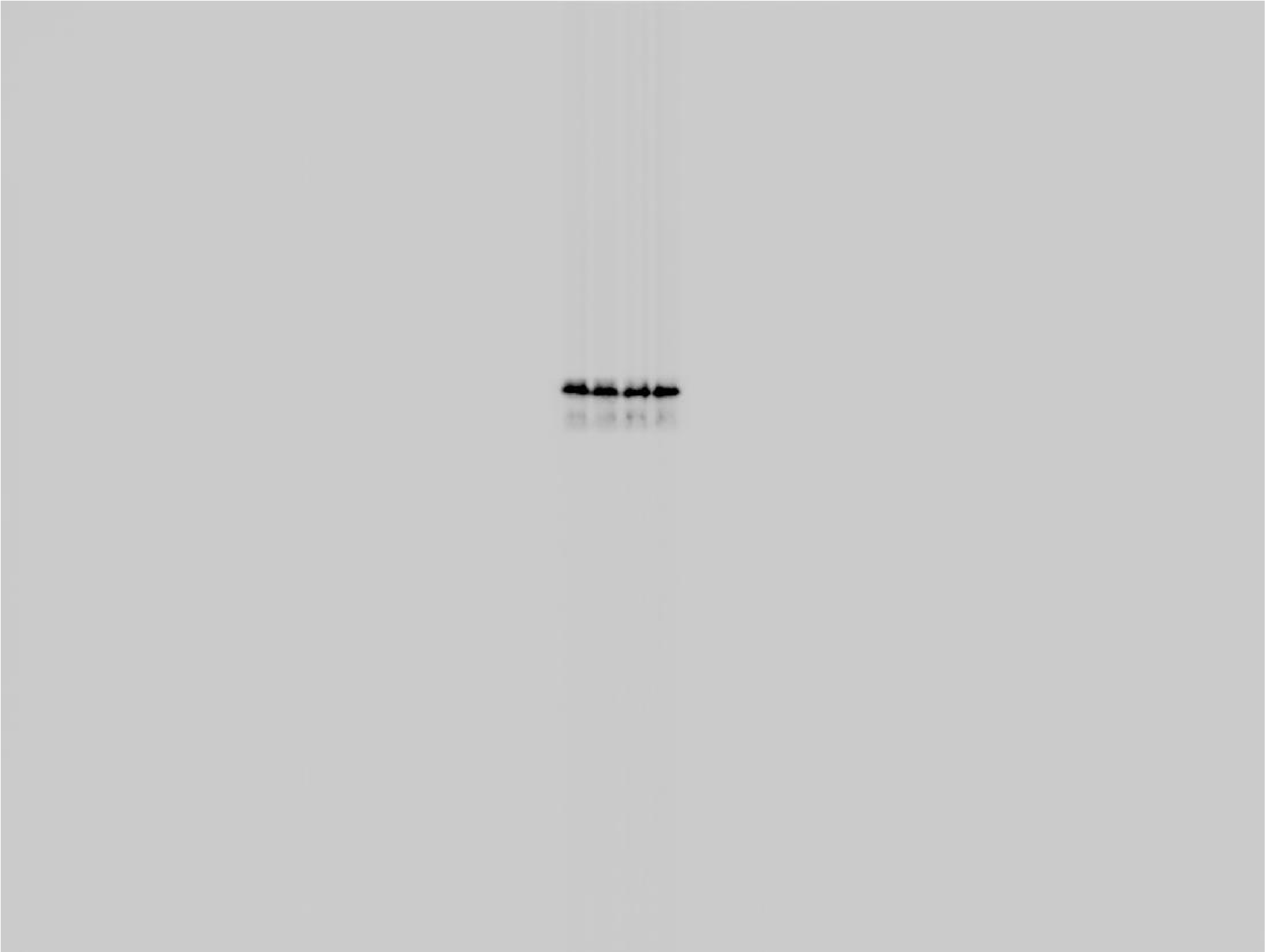

Figure 5A-H1703
GAPDH

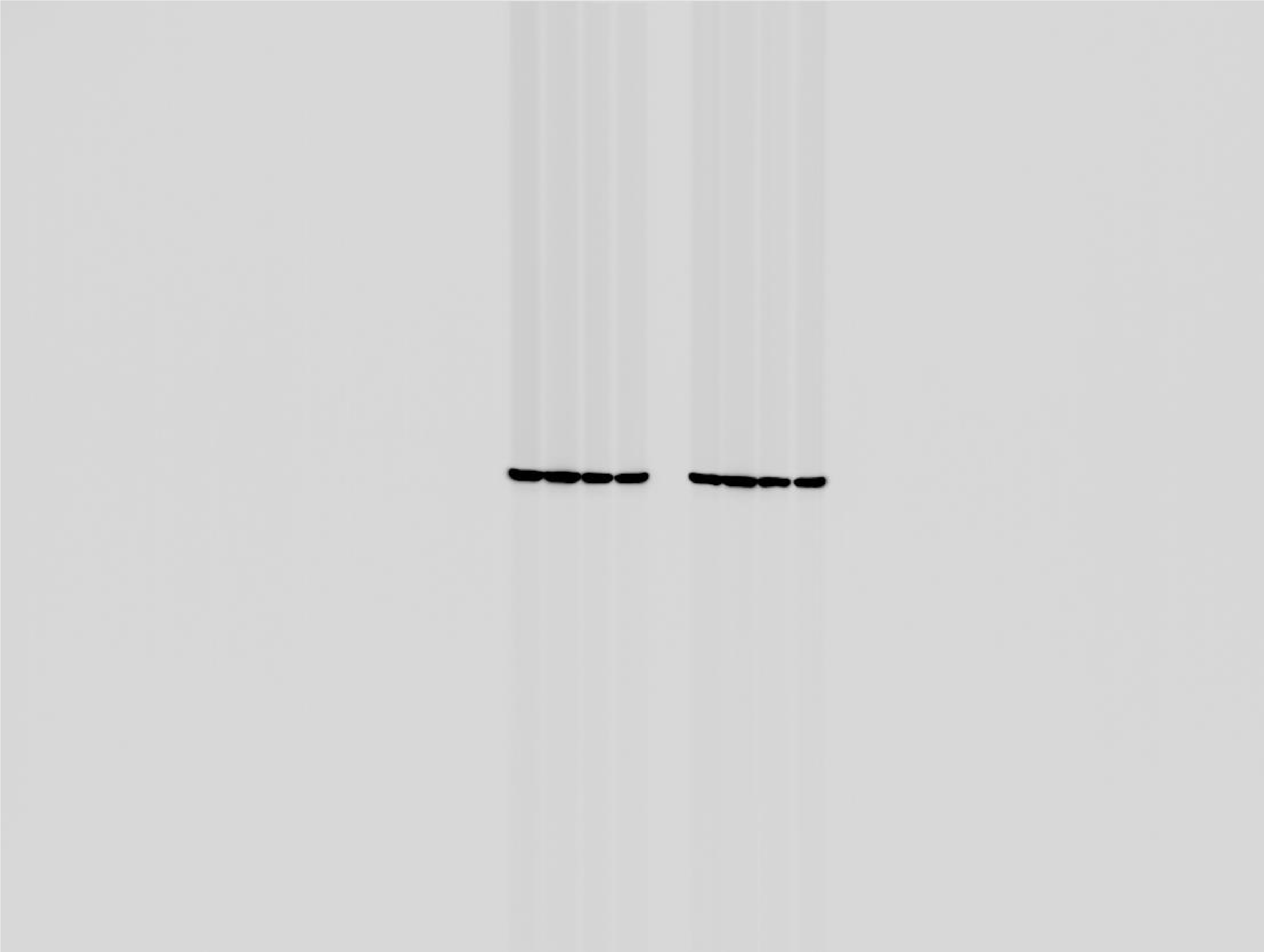

Figure 5A-H1703
mito DRP1

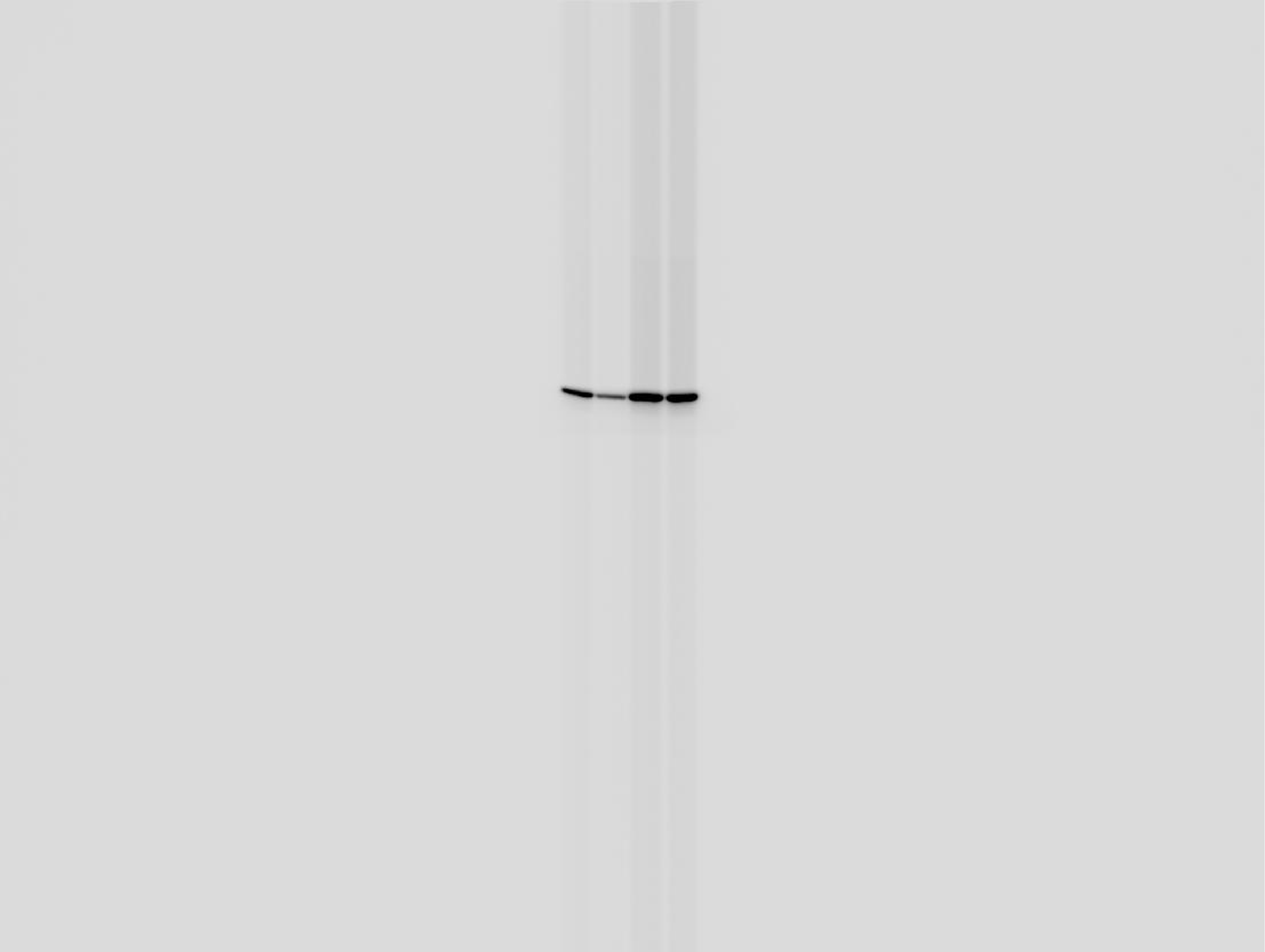

Figure 5A-H1703
mito MFN1

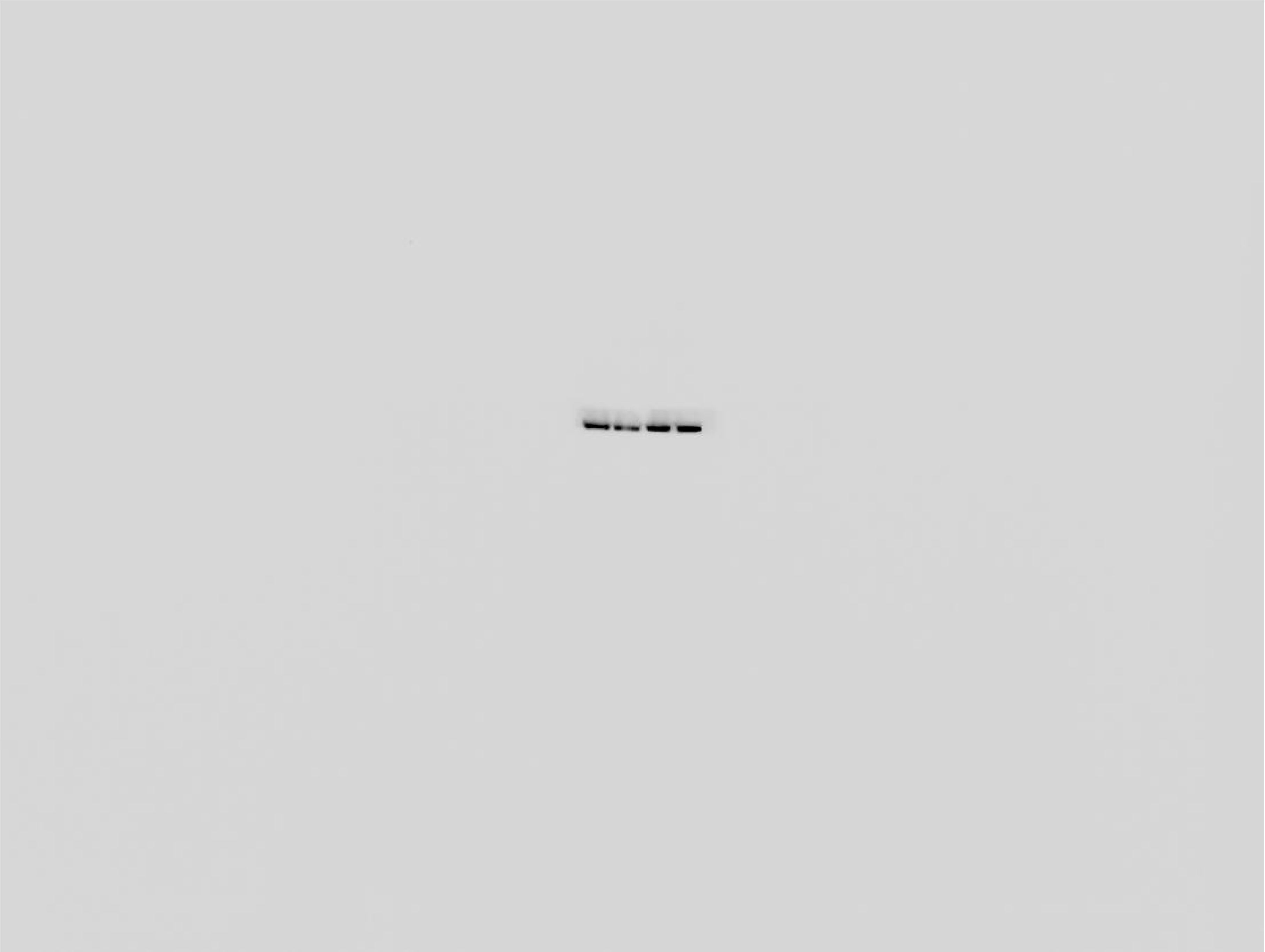

Figure 5A-H1703
VDAC

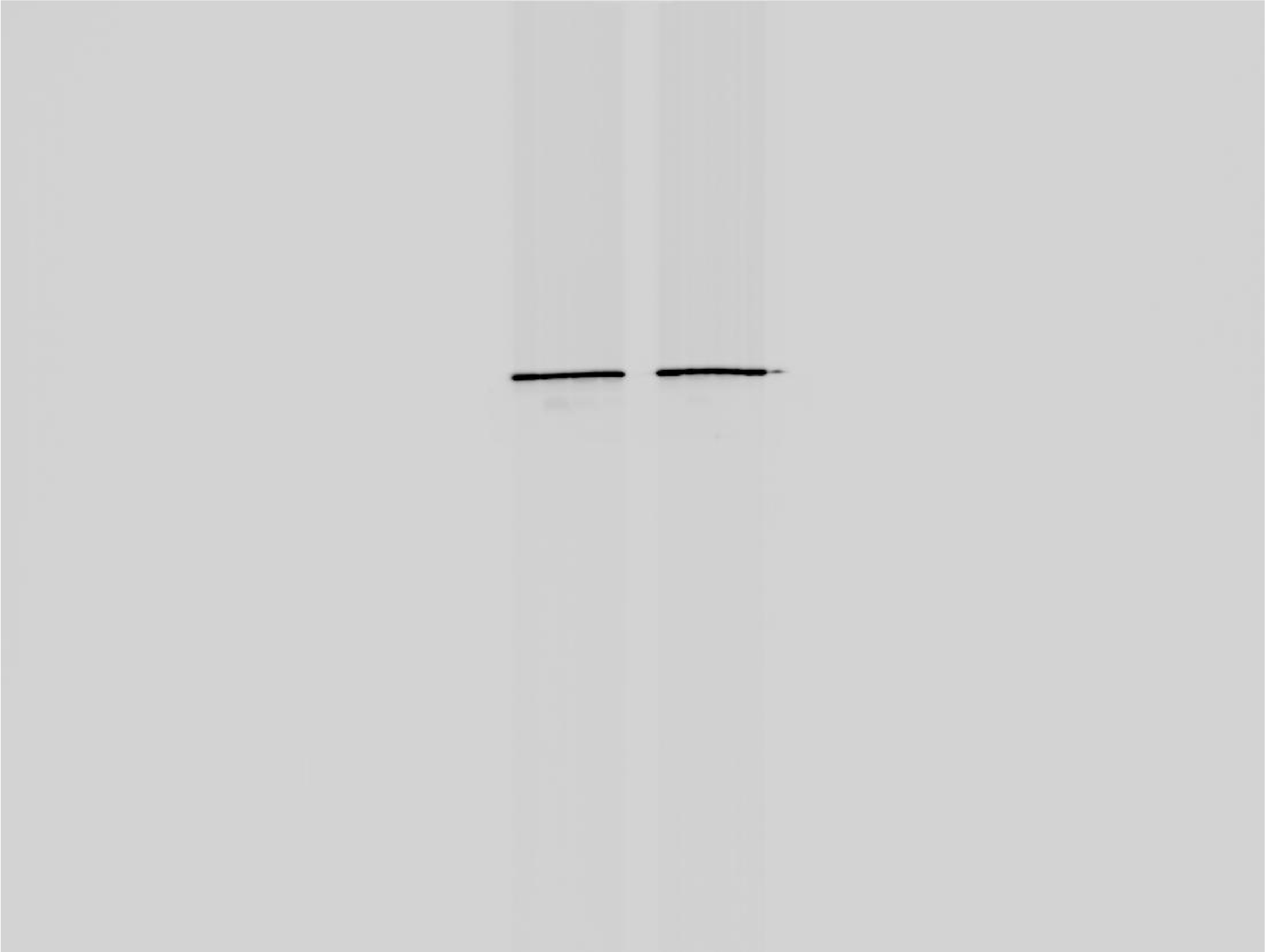

Figure 5B-H838 H1703
PINK

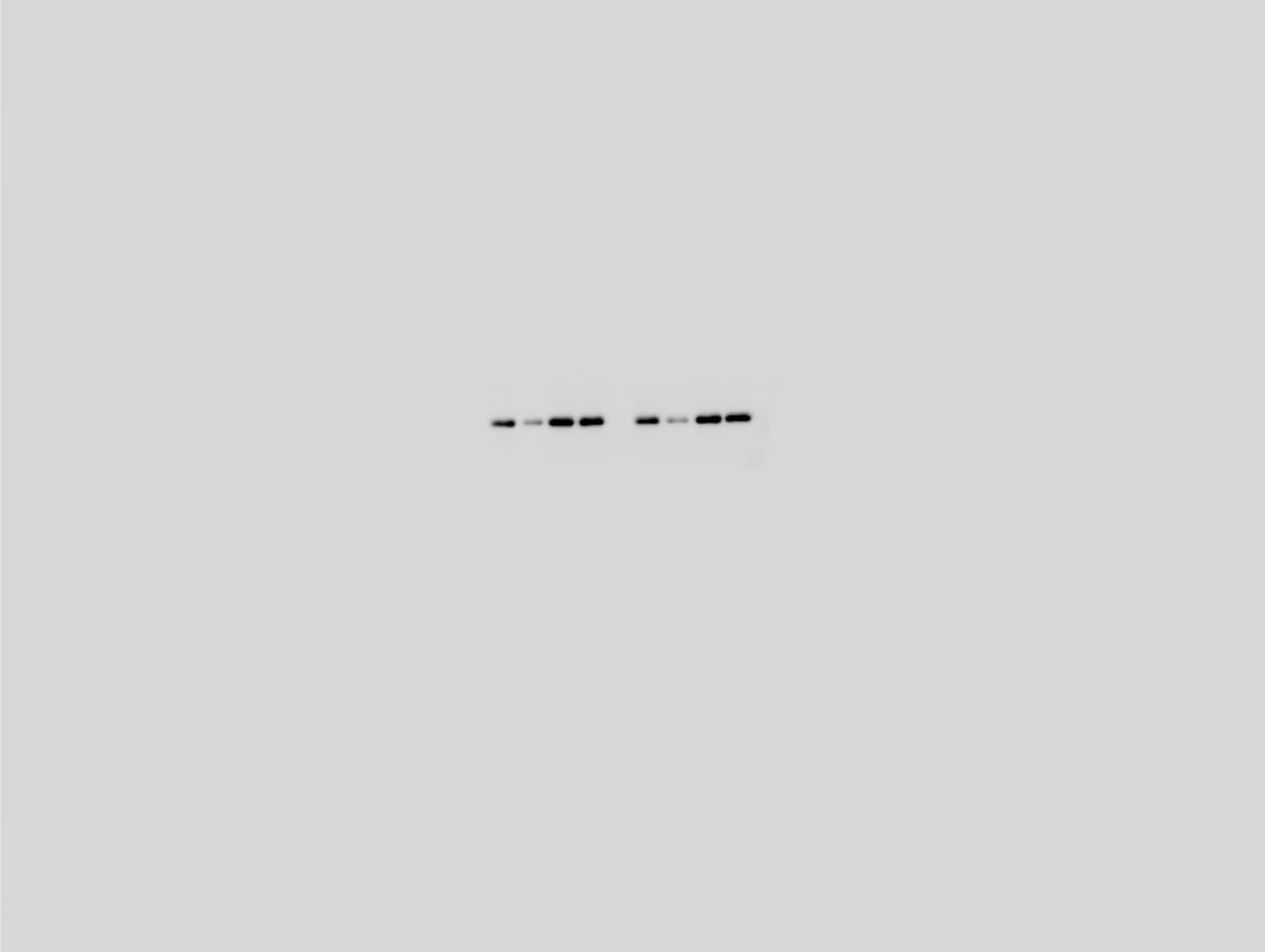

Figure 5B-H838 H1703
PARKIN

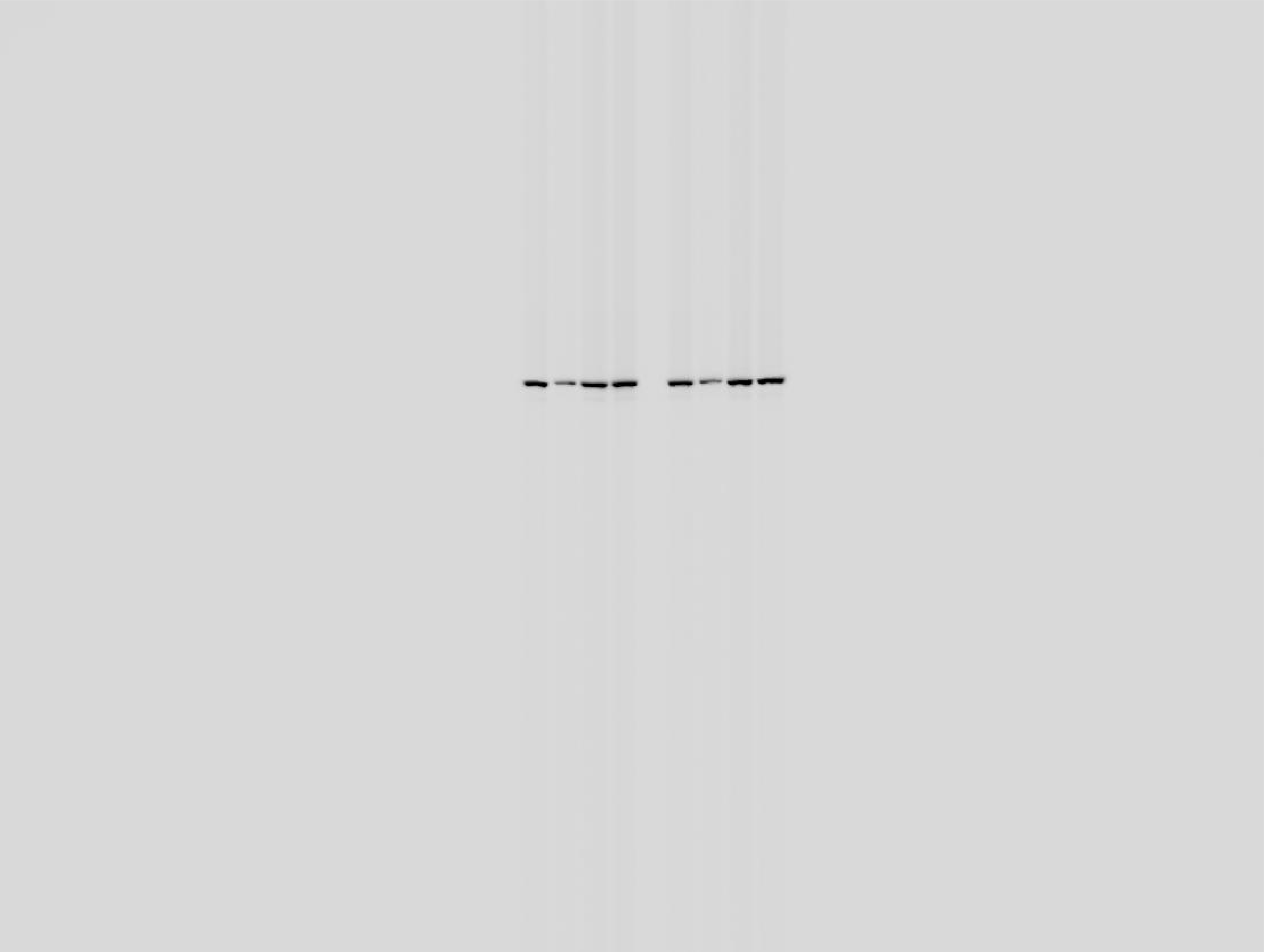

Figure 5B-H838 H1703
PGC-1α

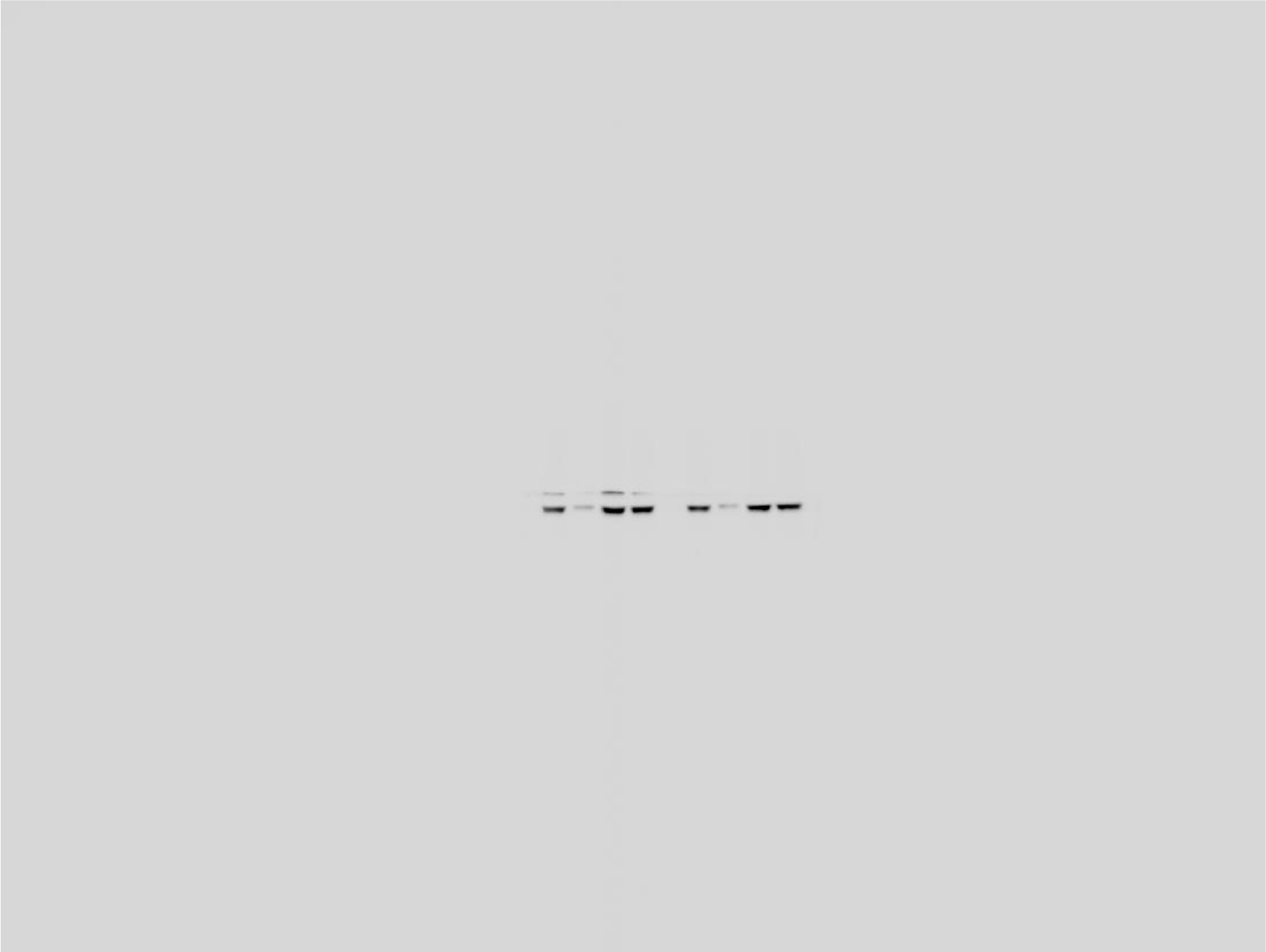

Figure 5B-H838 H1703
TFAM

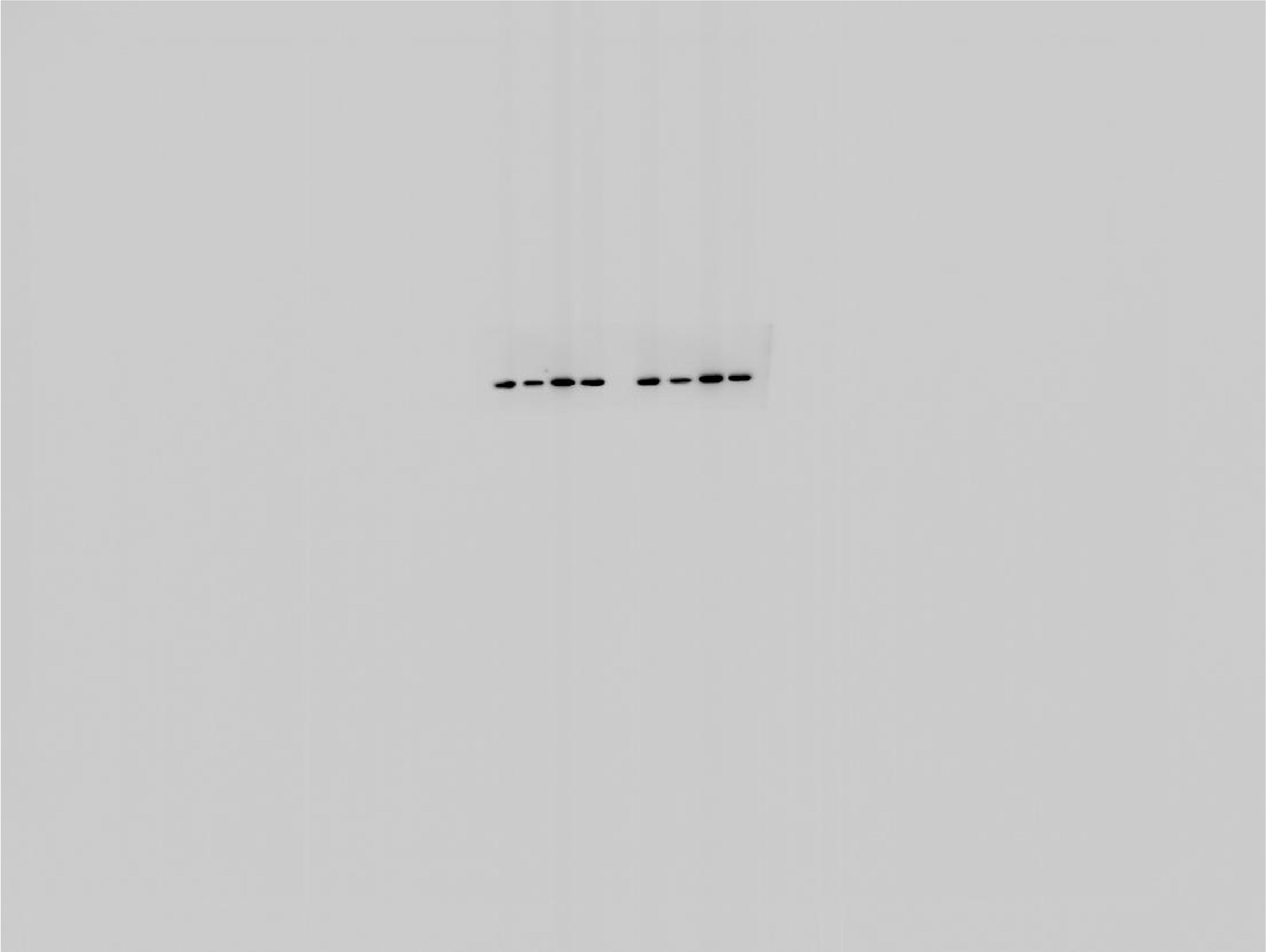

Figure 5B-H838

GAPDH

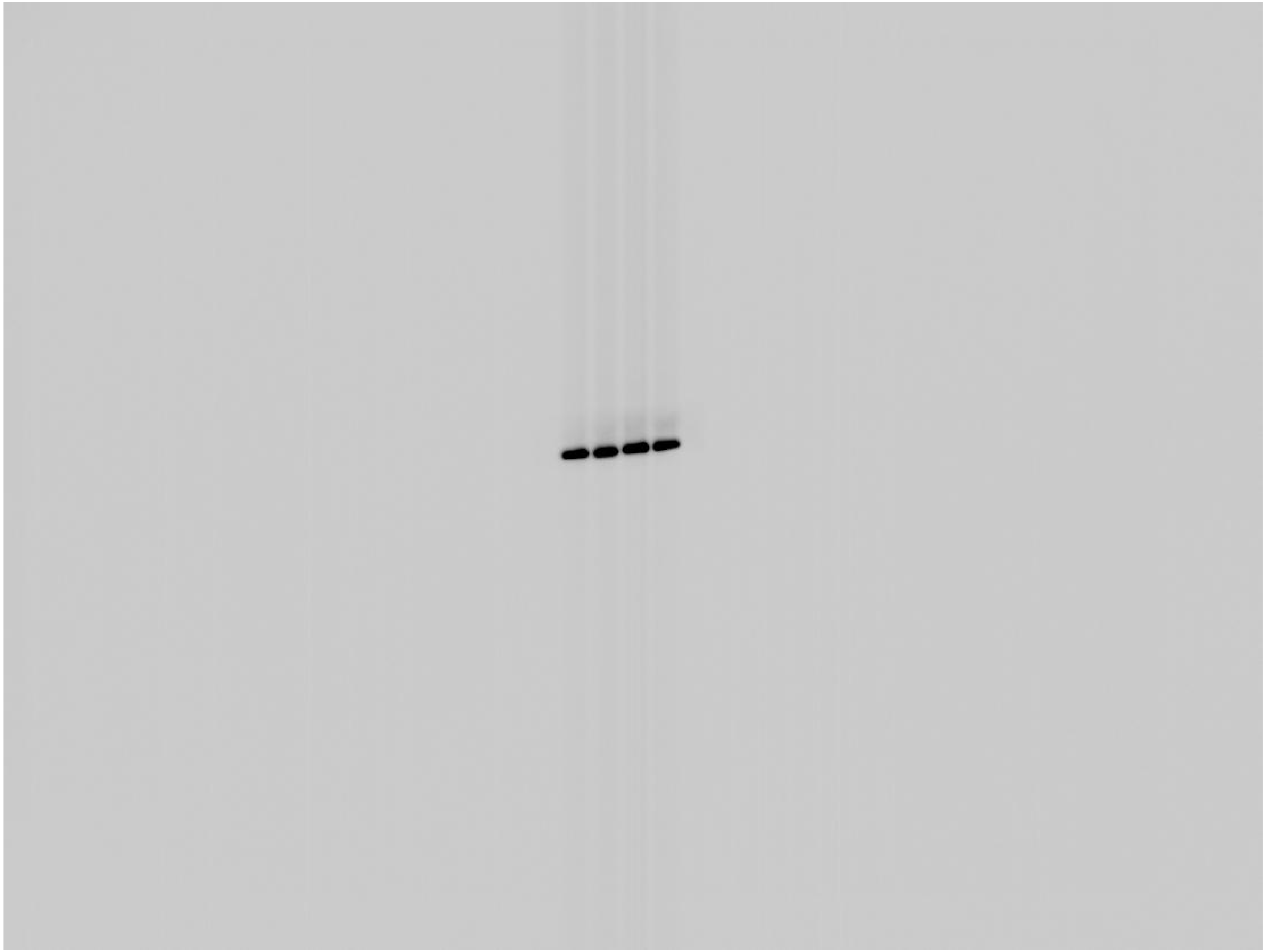

Figure 5B-H1703
GAPDH

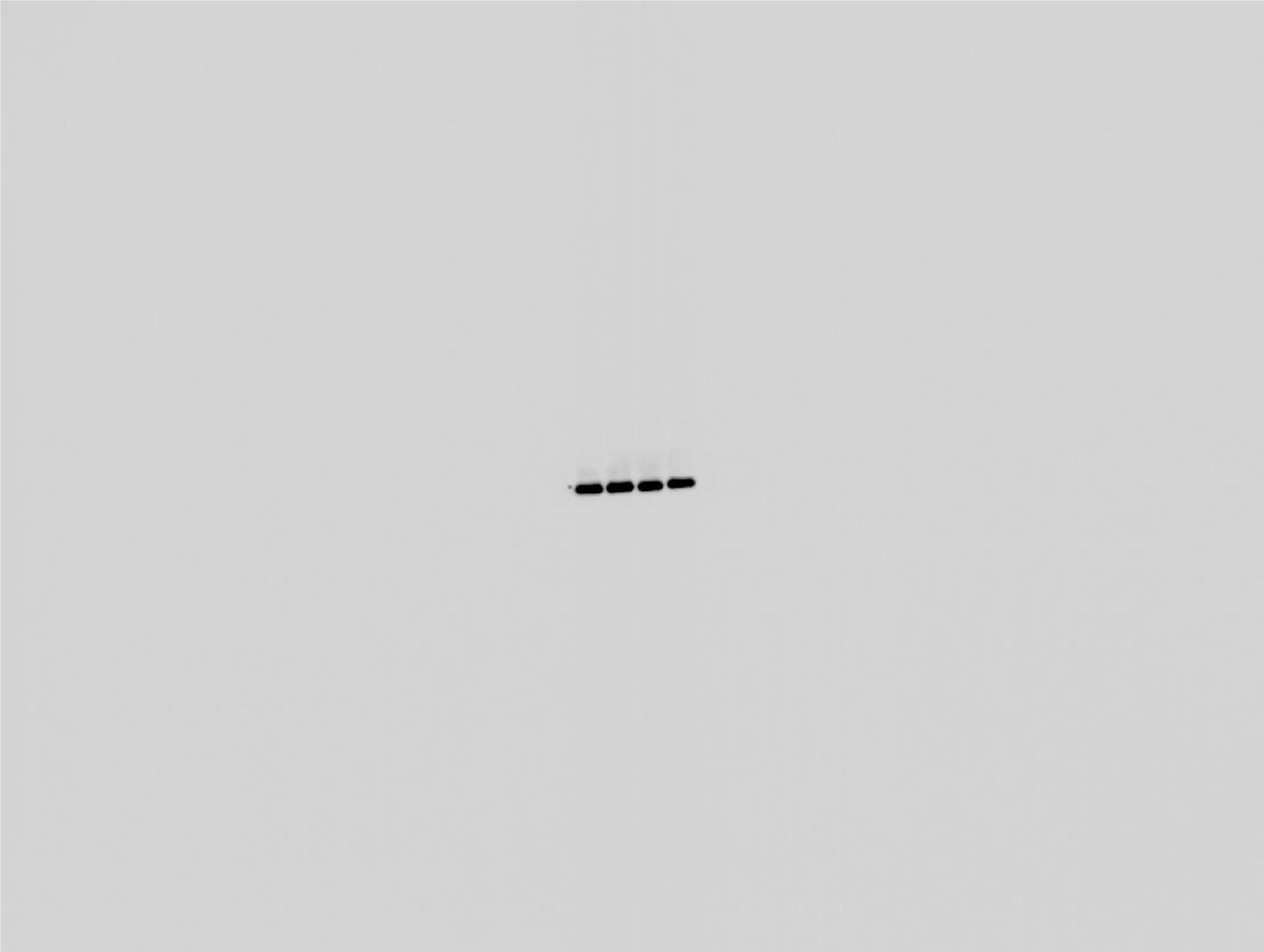

Figure S1B-COX7A1

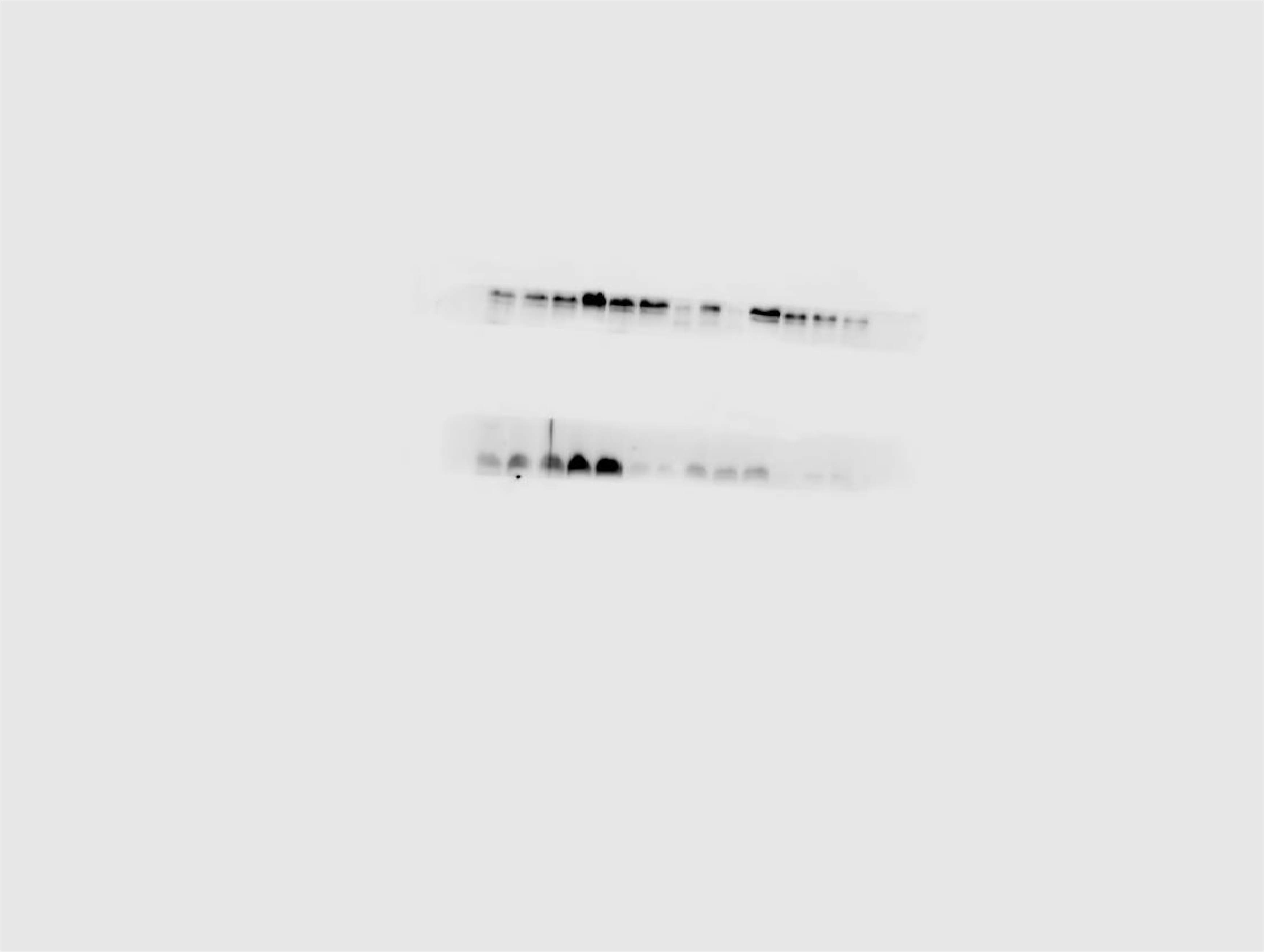

Figure S1B-GAPDH

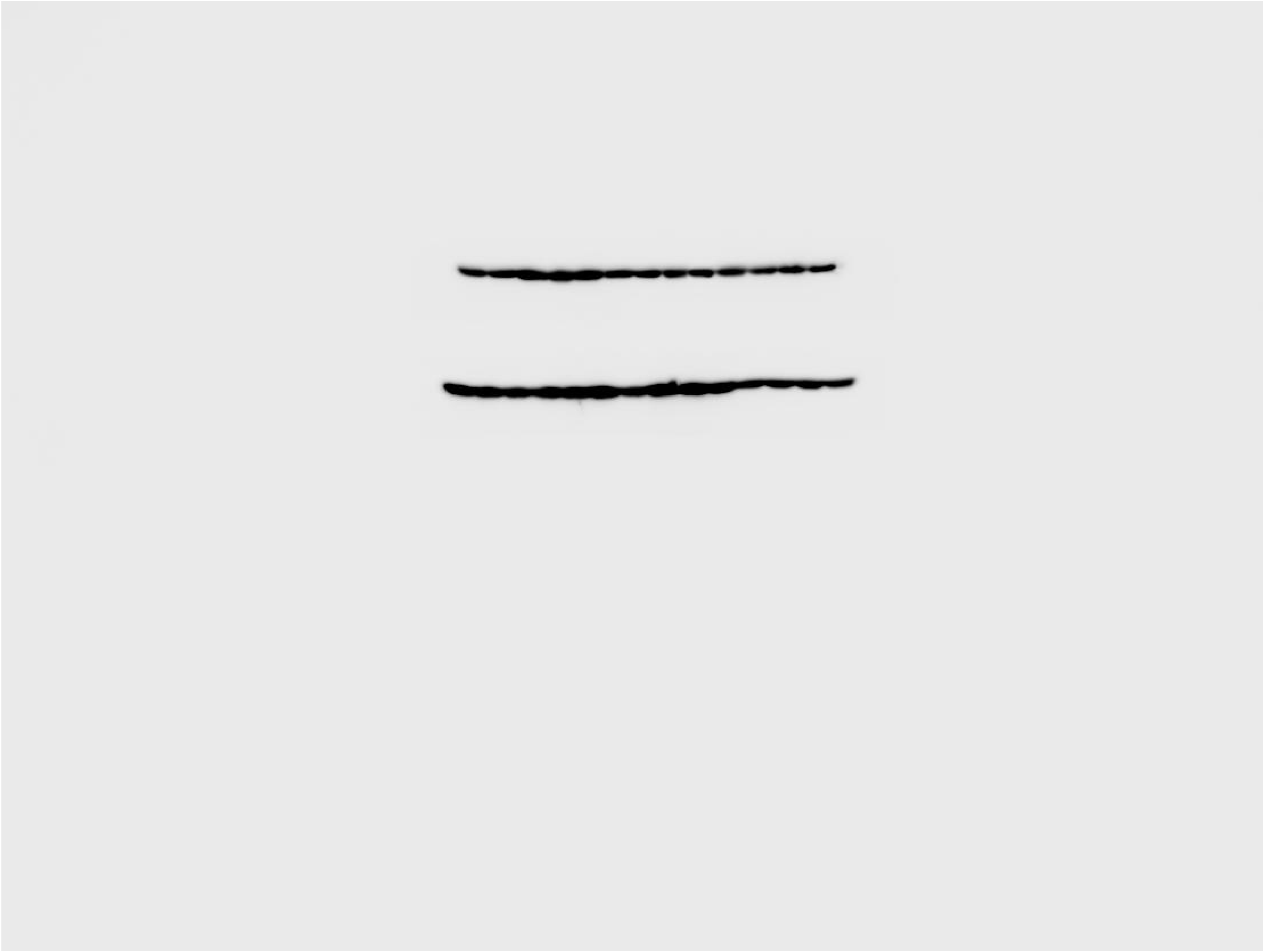

Figure S3A-p62

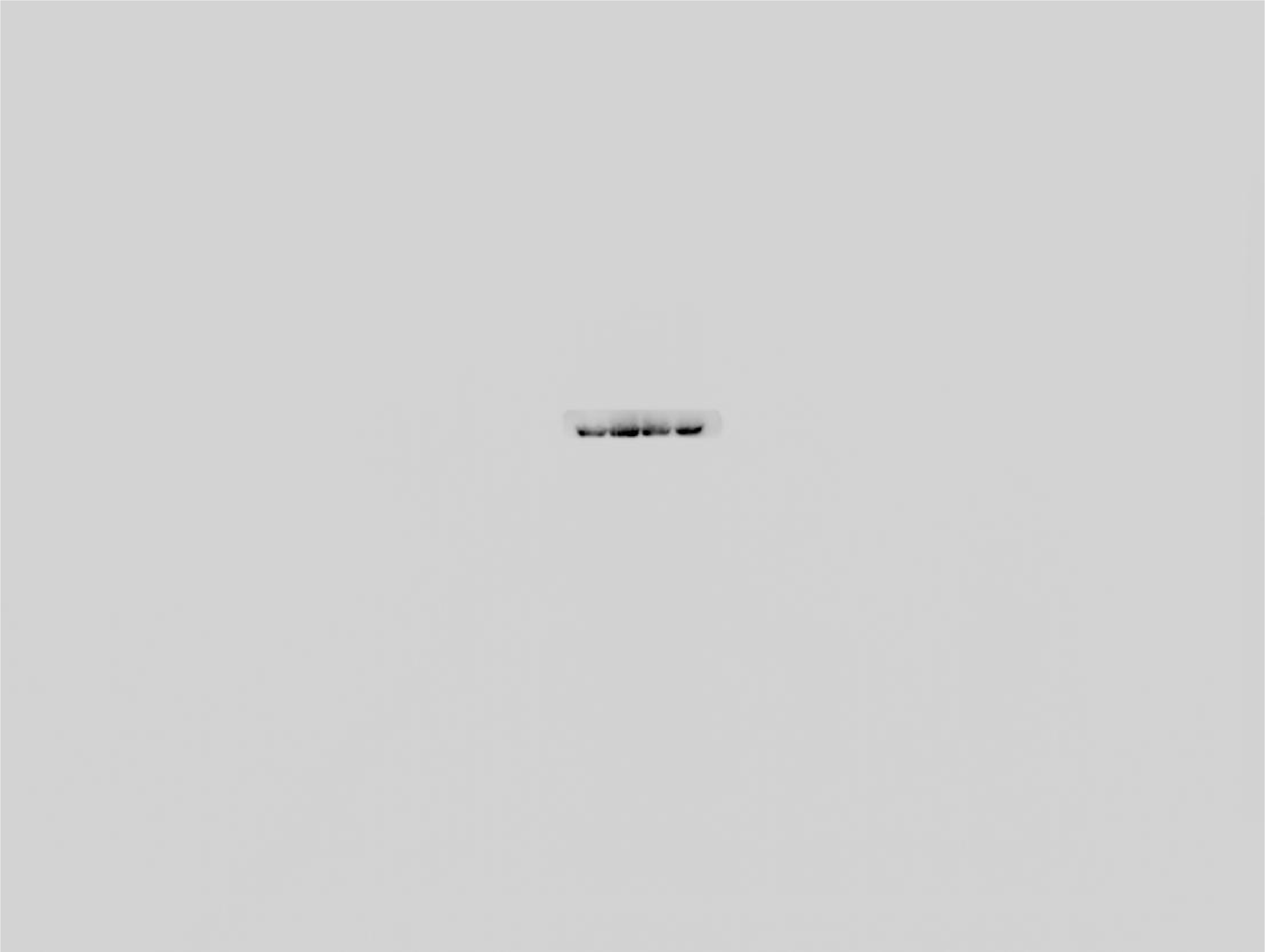

Figure S3A-LC3

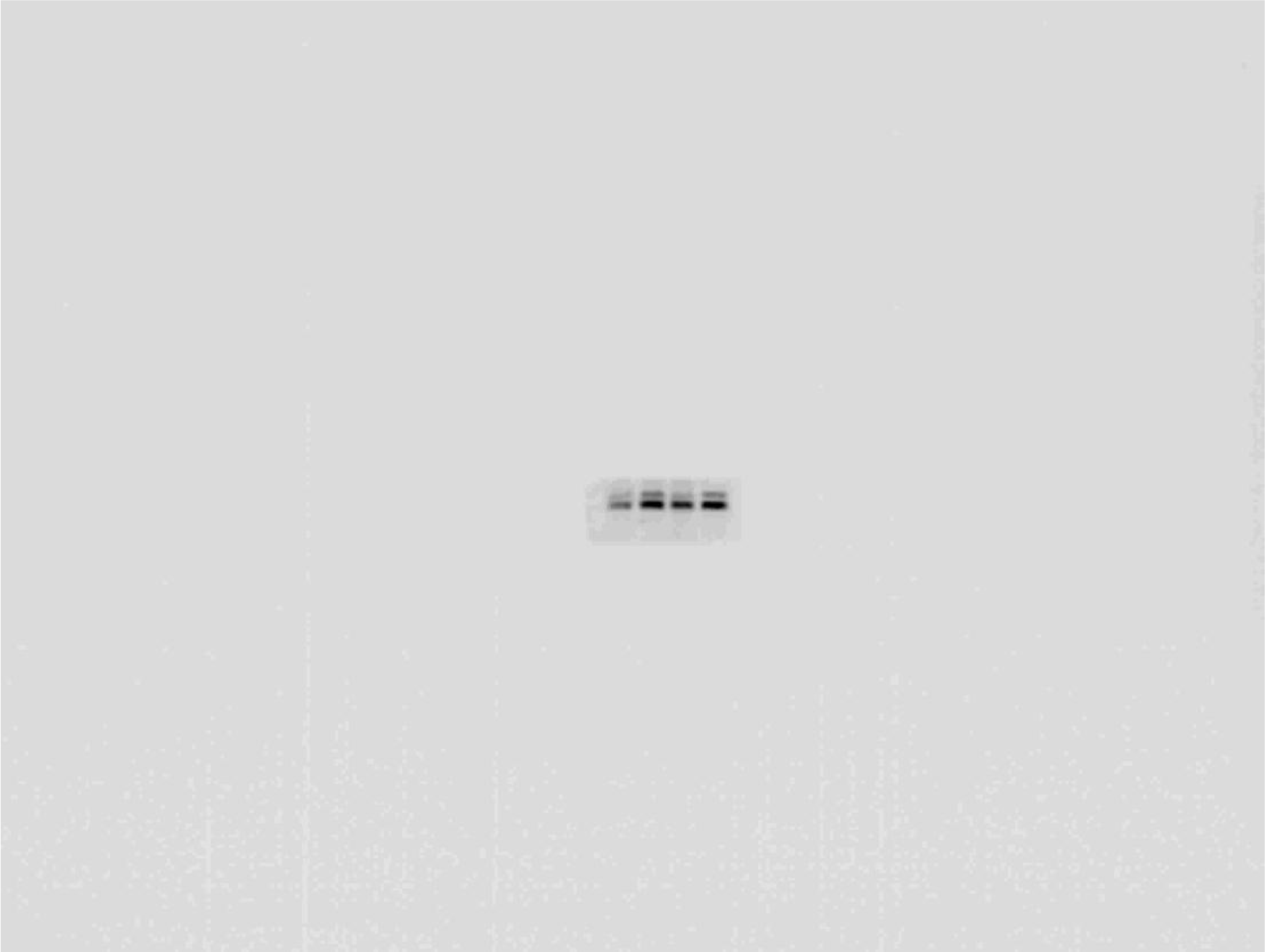

Figure S3A-GAPDH

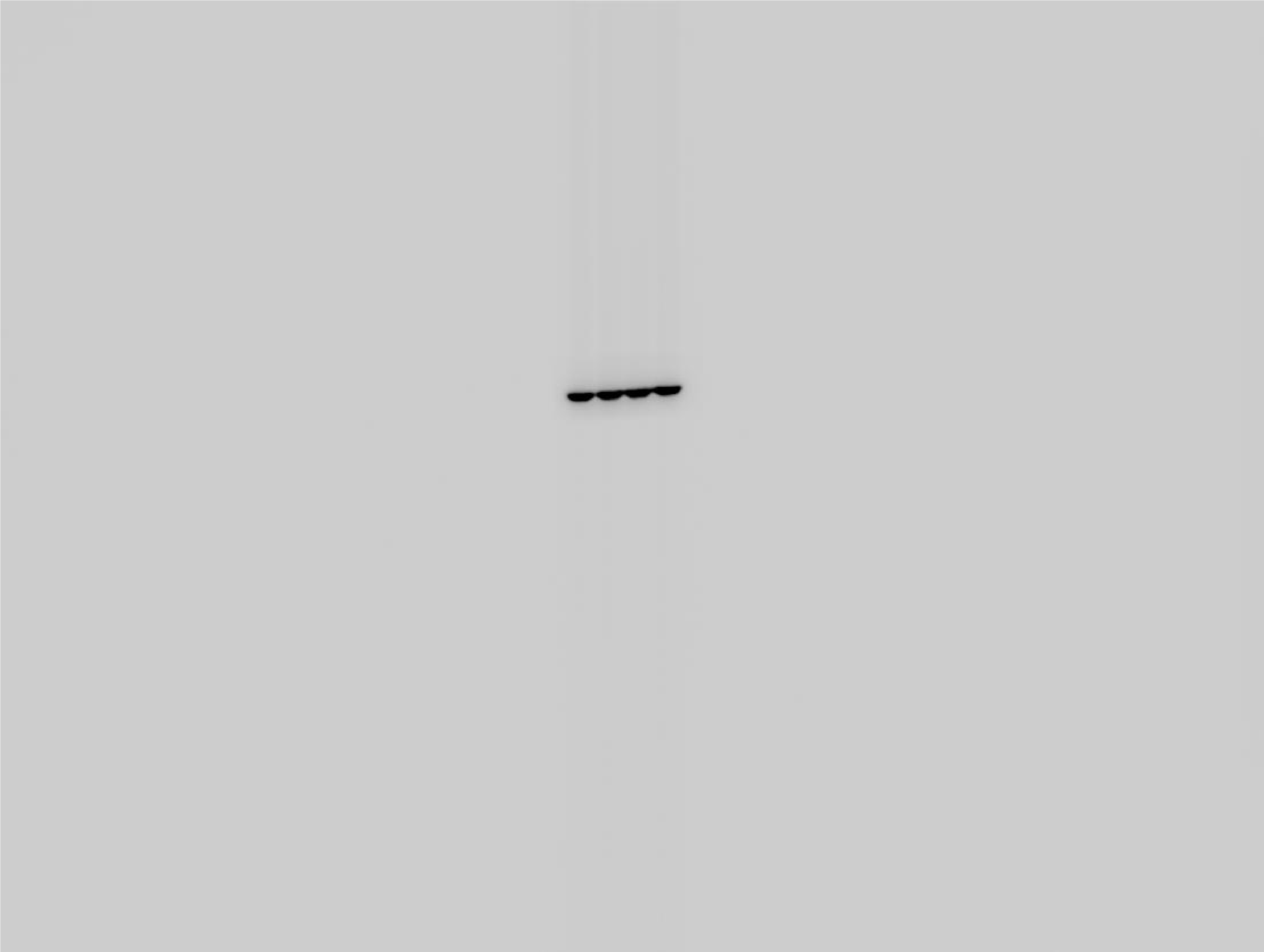

Figure S4B-TIM23
GAPDH

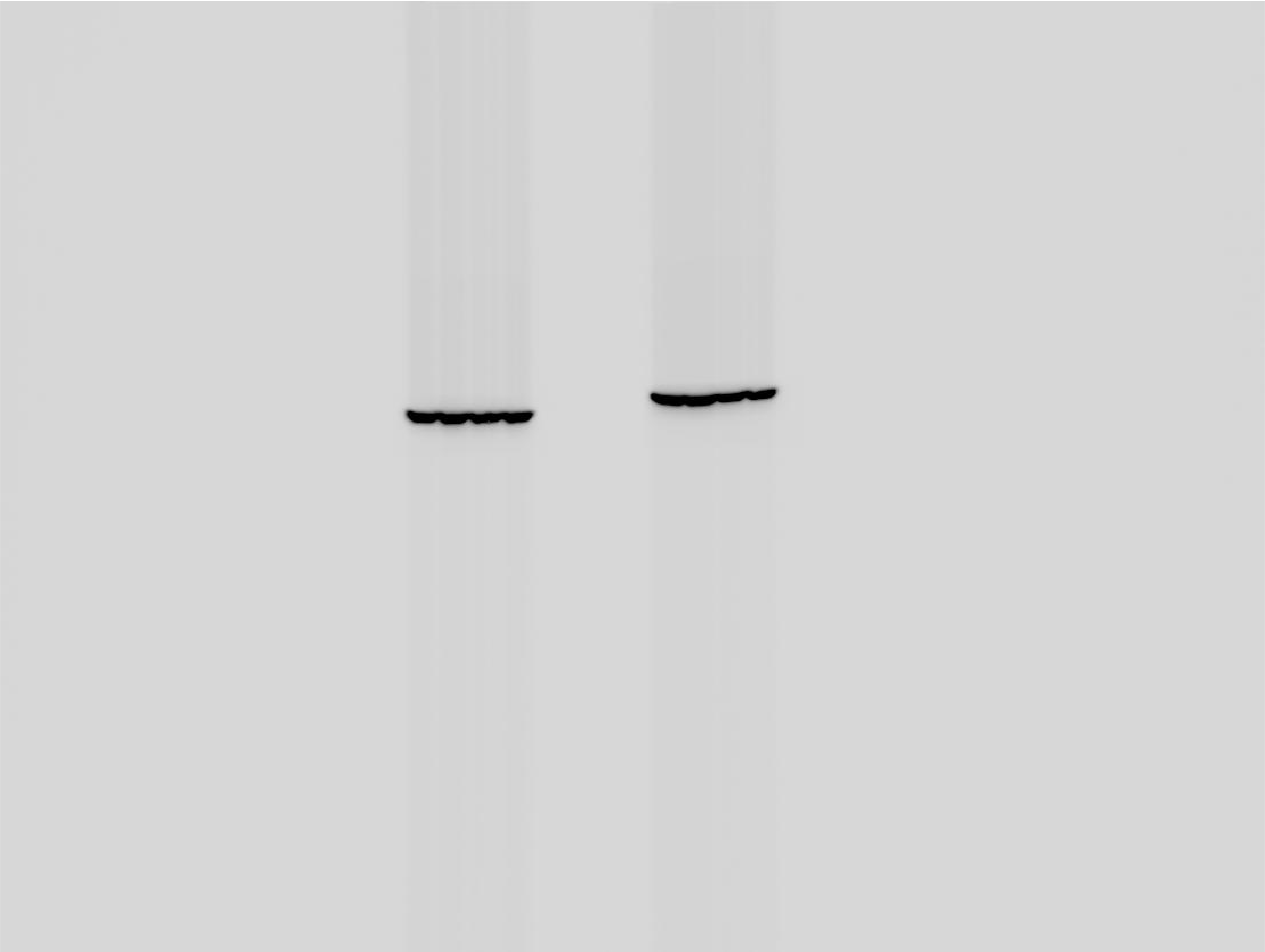

Figure S4B-TOM20

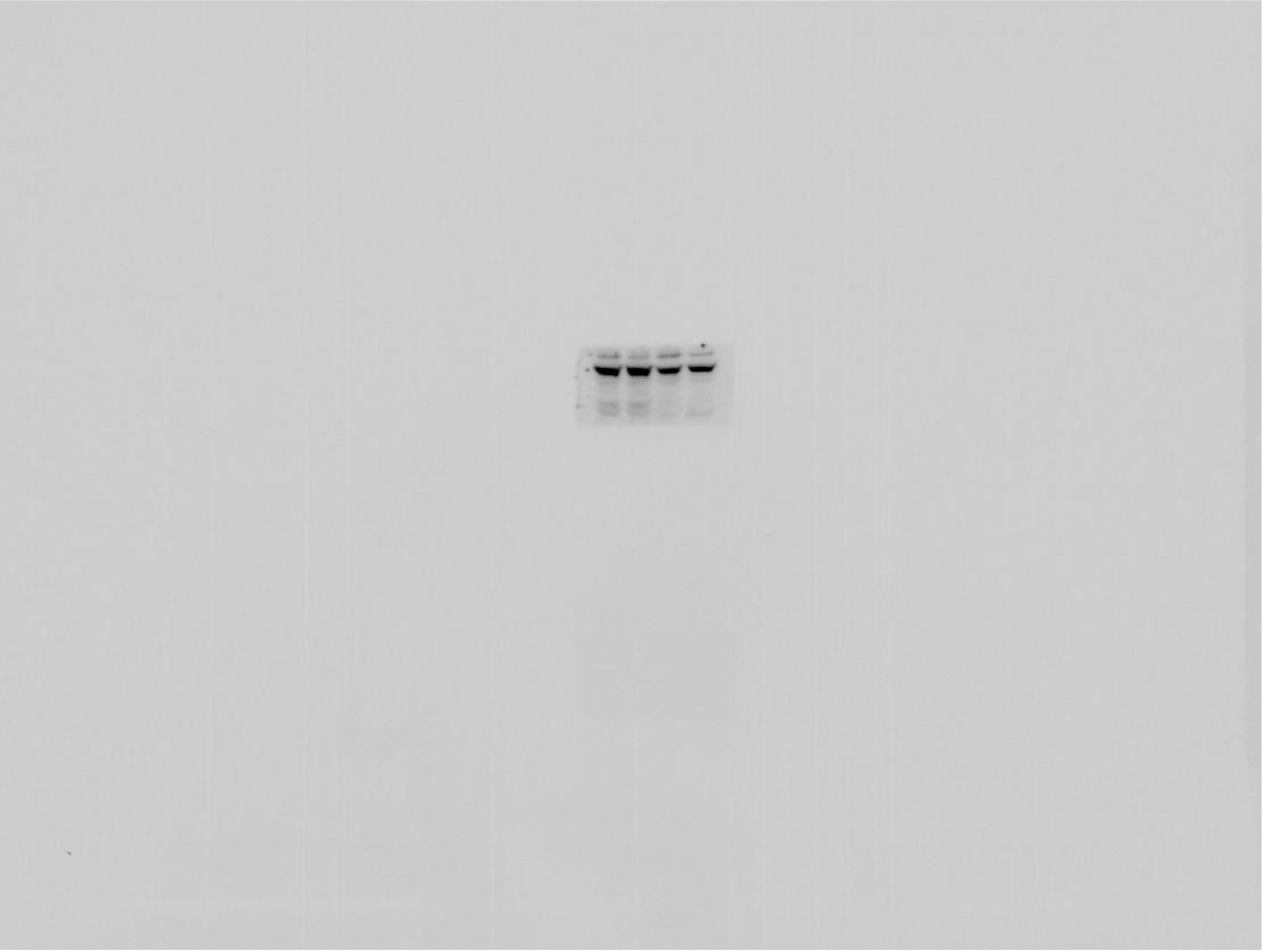

Supplement: Supplementary file 1 — Supplementary Figures [file 41419_2022_5430_MOESM1_ESM.pdf]
